# An Evaluation of Blood Compatibility of Silver Nanoparticles

**DOI:** 10.1038/srep25518

**Published:** 2016-05-05

**Authors:** He Huang, Wenjia Lai, Menghua Cui, Ling Liang, Yuchen Lin, Qiaojun Fang, Ying Liu, Liming Xie

**Affiliations:** 1 Key Laboratory of Standardization and Measurement for Nanotechnology of Chinese Academy of Sciences, CAS Center for Excellence in Nanoscience, National Center for Nanoscience and Technology, Beijing 100190, P. R. China; 2 Key Laboratory for Biomedical Effects of Nanomaterials and Nanosafety of Chinese Academy of Sciences, CAS Center for Excellence in Nanoscience, National Center for Nanoscience and Technology, Beijing 100190, P. R. China; 3 College of Chemistry and Chemical Engineering, Lanzhou University, Lanzhou, 730000, P. R. China

## Abstract

Silver nanoparticles (AgNPs) have tremendous potentials in medical devices due to their excellent antimicrobial properties. Blood compatibility should be investigated for AgNPs due to the potential blood contact. However, so far, most studies are not systematic and have not provided insights into the mechanisms for blood compatibility of AgNPs. In this study, we have investigated the blood biological effects, including hemolysis, lymphocyte proliferation, platelet aggregation, coagulation and complement activation, of 20 nm AgNPs with two different surface coatings (polyvinyl pyrrolidone and citrate). Our results have revealed AgNPs could elicit hemolysis and severely impact the proliferation and viability of lymphocytes at all investigated concentrations (10, 20, 40 μg/mL). Nevertheless, AgNPs didn’t show any effect on platelet aggregation, coagulation process, or complement activation at up to ~40 μg/mL. Proteomic analysis on AgNPs plasma proteins corona has revealed that acidic and small molecular weight blood plasma proteins were preferentially adsorbed onto AgNPs, and these include some important proteins relevant to hemostasis, coagulation, platelet, complement activation and immune responses. The predicted biological effects of AgNPs by proteomic analysis are mostly consistent with our experimental data since there were few C3 components on AgNPs and more negative than positive factors involving platelet aggregation and thrombosis.

In recent years, nanoparticles (NPs) defined as materials whose main components have one dimension between 1 and 100 nanometer by the European Commission, have been increasingly used in many fields of our daily life such as consumer goods, science, engineering, medicine due to their special properties^1^. The applications of NPs are particularly popular in the current research areas of material science as well as biomedicine due to their larger surface area-to-volume ratios resulting in better reactivity^2^.

Silver materials, such as metal silver and silver salts, are powerful antimicrobial agents in medicine for centuries^3^,^4^. Silver nanoparticles (AgNPs), with broad antimicrobial spectrum as well as high efficacy against specific bacteria, have been widely used in consumer products^5^,^6^. It has also been reported that AgNPs have anti-virus, anti-inflammation, anti-biofilm activities and enhance wound healing^7^,^8^,^9^,^10^. With the small size, AgNPs may translocate into the circulatory system through dermal contact, inhalation, ingestion systemic administration or even injection^11^,^12^,^13^. Once entering the circulatory system, pristine nanoparticles will come into contact with blood cells as well as plasma proteins and potentially trigger pathophysiologic processes. Hence, the blood compatibility of AgNPs needs to be carefully investigated. According to ISO-10993-4, in principle, for evaluation of the interaction of medical devices with blood, thrombosis, coagulation, platelets, haematology, complement system should be investigated^14^.

Hemolysis (or hameolysis) is the rupture of erythrocytes (red blood cells) and the release of their contents (*e.g.*, hemoglobin) into the surroundings^15^, leading to anaemia, jaundice and renal failure^16^. Platelets, also known as thrombocytes, have been found to play a key role in mediating innate immunity^17^. The loss of platelet function which is crucial to the primary haemostasis can cause hemorrhagic or thrombotic disorders^13^. As the secondary haemostasis, plasma coagulation cascade is responsible for blood clotting and can be activated by platelets^18^. Complement activation as a defense reaction is potentially initiated by blood contacting with pathological invaders^19^.

Although many studies have been performed on the biological effects of AgNPs in blood, the materials used were different from each other in sizes and coatings, which results in great difficulty for comparisons. Moreover, they are lack of suitable controls to evaluate the real reason of AgNPs’ toxicity. Besides, most studies are not systematic and few have been provided insights into the mechanisms, which impedes a comprehensive evaluation of AgNPs’ blood compatibility. So far, it has been reported that due to the large surface area, a significant increase in *in vitro* hemolysis was observed with AgNPs compared with micron-sized particles^20^. AgNPs could prevent platelet responses as evidenced by the similar inhibitory effects of AgNPs of different sizes (13–15 nm, 30–35 nm and 40–45 nm) on platelet aggregation^21^. The interference of AgNPs on plasma coagulation, similar to platelet aggregation, is different depending on various sizes, coatings and concentrations^18^,^22^,^23^,^24^. For the effects of AgNPs on the process of complement activation, reports were limited^25^.

In this study, we have systematically investigated hemolysis, platelet aggregation, coagulation, lymphocyte proliferation and the activation of complement system after the treatment with 20 nm AgNPs coated with polyvinyl pyrrolidone (AgNP-PVP-20) and citrate (AgNP-CIT-20). Since plasma corona of nanoparticles has implications on biological processes that control haemostasis, thrombosis and inflammatory responses^26^,^27^,^28^,^29^,^30^. AgNPs coronas were characterized by label-free quantitative mass spectrometry analysis. The proteomic analysis showed that there were few complement 3 (C3) components on AgNPs and more negative than positive factors involving platelet aggregation and thrombosis. That might be responsible for our observations that AgNP-PVP-20 and AgNP-CIT-20 had no significant effect on plasma coagulation, platelet aggregation and complement activation.

## Results

### Characterization of AgNPs

**Figure 1:**
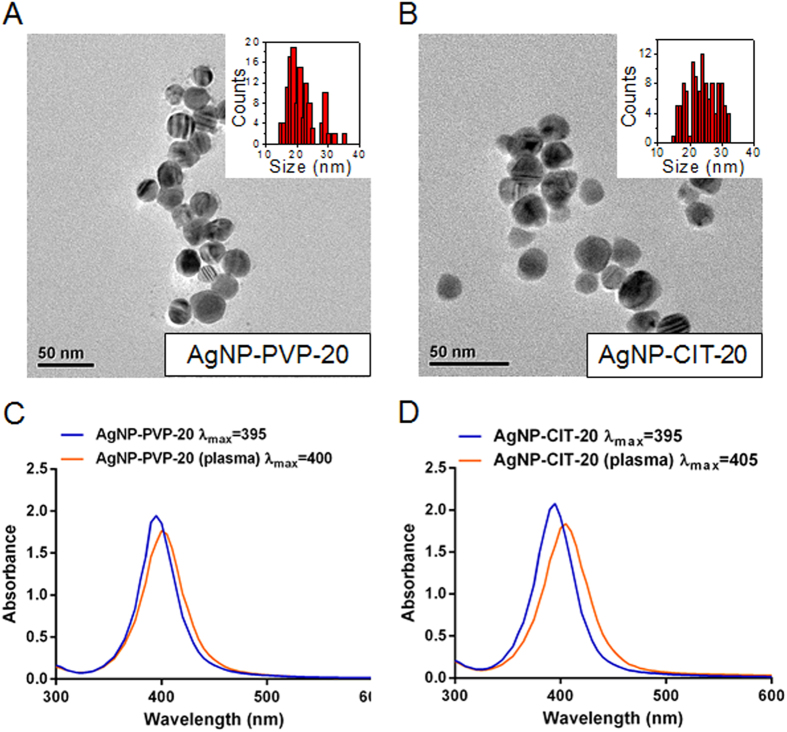
Physicochemical characterization of AgNPs. The representative TEM images and size distribution histograms (insets) of AgNP-PVP-20 (**A**) and AgNP-CIT-20 (**B**). UV-Vis absorption spectra in water and after interacting with plasma for AgNP-PVP-20 (**C**) and AgNP-CIT-20 (**D**), respectively. Scale bars represented 50 nm.

20 nm polyvinyl pyrrolidone (PVP) -coated AgNPs (AgNP-PVP-20) and 20 nm citrate-coated AgNPs (AgNP-CIT-20) were used in this study. Transmission electron microscopy (TEM) imaging showed that AgNPs were closed to spherical in shape and the average individual sizes were 21.6 ± 4.8 nm for AgNP-PVP-20 and 24.3 ± 4.5 nm for AgNP-CIT-20, respectively (Fig. 1A,B). UV-Vis absorption spectra of both AgNPs displayed maximum absorption peak around 395 nm in water (Fig. 1C,D). In plasma medium, the adsorption peak of both AgNPs showed a red shift of 5–10 nm, an indication of protein adsorption on AgNPs^23^.

The hydrodynamic size (d_H_) and zeta potential (ζ) of AgNPs in various media used in this study (water, plasma, Dulbecco’s Phosphate-Buffered Salines DPBS and RPMI) were summarized in Table 1. AgNP-CIT-20 dispersed well with no aggregation in water, while AgNP-PVP-20 slightly aggregated (d_H_ of 58.6 ± 2.4 nm). Both AgNPs appeared to slightly aggregate in plasma (d_H_ of 50–60 nm) and obviously aggregated in DPBS and RPMI-1640 culture medium (d_H_ lagrer than 90 nm). The potentials of both AgNPs in all media were negative. AgNPs in DPBS and RPMI-1640 showed negative ζ −6.4 and −6.6 mV for AgNP-PVP-20 and −9.9 and −16.1 mV for AgNP-CIT-20, which are less than that in other media (<−20 mV).

### Hemolysis

**Figure 2:**
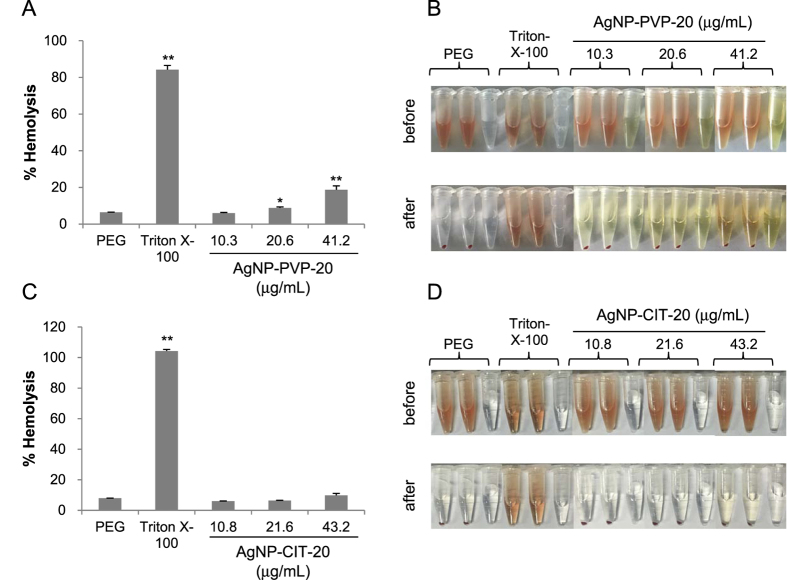
Hemolytic activity of AgNPs. Percentage of hemolysis induced by AgNP-PVP-20 (**A**) and AgNP-CIT-20 (**C**), respectively. Visual inspection of the tubes containing diluted total blood (TBHd) after exposure to AgNP-PVP-20 (**B**) or AgNP-CIT-20 (**D**) for 3 h before or after centrifugation. PEG and Triton-X-100 were respectively used as negative control and positive control. *p < 0.05, **p < 0.01, significantly different from negative control.

All materials enter the blood get in contact with red blood cells (RBC). To assess the impact of AgNPs on erythrocyte, hemolysis test was performed by spectrophotometric measurement of hemoglobin release after exposure to various concentrations of 20 nm AgNPs. The performance of hemolysis assay was tested by the negative control polyethylene glycol (PEG) and positive control Triton-X-100 (Fig. 2). The hemolytic activity of AgNP-PVP-20 and AgNP-CIT-20 showed dose-dependent hemolysis, in which AgNP-PVP-20 was more potent than AgNP-CIT-20. At the concentration of 40 μg/mL, AgNP-PVP-20 caused ~19% hemolysis (Fig. 2A,B) while AgNP-CIT-20 only led to 10% hemolysis (Fig. 2C,D).

### The proliferation of peripheral lymphocytes

**Figure 3:**
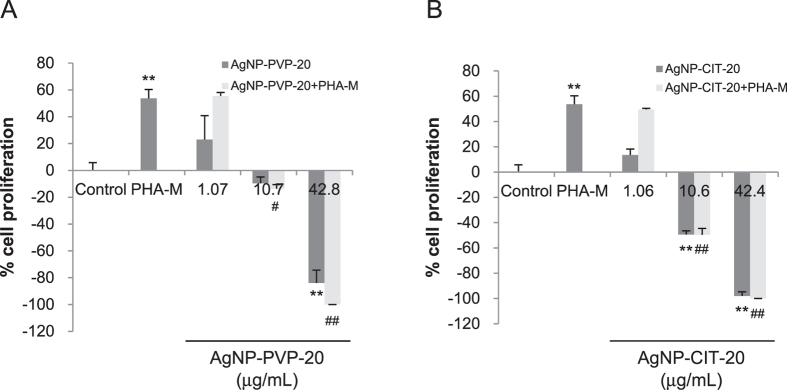
Lymphocyte proliferation after AgNPs treatment. PBMCs were treated with different concentrations of AgNP-PVP-20 (**A**) or AgNP-CIT-20 (**B**) for 3 days, and then measured by MTS assay. RPMI-1640 and PHA-M were used as negative control and positive control, respectively. **p < 0.01, significantly different from negative control. ^#^*p* < 0.05, ^##^*p* < 0.01, significantly different from positive control.

To further explore the safety of AgNPs in the bloodstream, lymphocyte proliferative responses were investigated to assess the effects of AgNPs on the basic immunological functions of human lymphocytes. Peripheral blood monocytes (PBMCs) were treated with different doses of PVP or citrate coated AgNPs for 72 h and measured by MTS assay. As shown in Fig. 3, comparing with negative control (RPMI-1640), positive control phytohemagglutinin (PHA-M) induced approximately 55% of lymphocyte proliferation. At the low concentration of about 1 μg/mL, both AgNPs could slightly promote less than 20% of lymphocyte proliferation without significant difference versus negative control (Fig. 3A,B). Moreover, both AgNPs with low concentration did not significantly suppress PHA-M-induced lymphocyte proliferation. Along with the increased concentrations up to ~40 μg/mL, both AgNP-PVP-20 and AgNP-CIT-20 exhibited strong inhibition to lymphocyte proliferation. These results indicated that at the concentration of 10–40 μg/mL, AgNP-PVP-20 and AgNP-CIT-20 were quite cytotoxic to human lymphocytes. In addition, since the stock solution of AgNP-CIT-20 was in 2 mM citrate solution, we also detected the effects of aqueous citrate with the corresponding concentrations. The result showed that lymphocyte proliferation was not disturbed by the existence of citrate (Fig. S1A).

### Platelet aggregation

**Figure 4:**
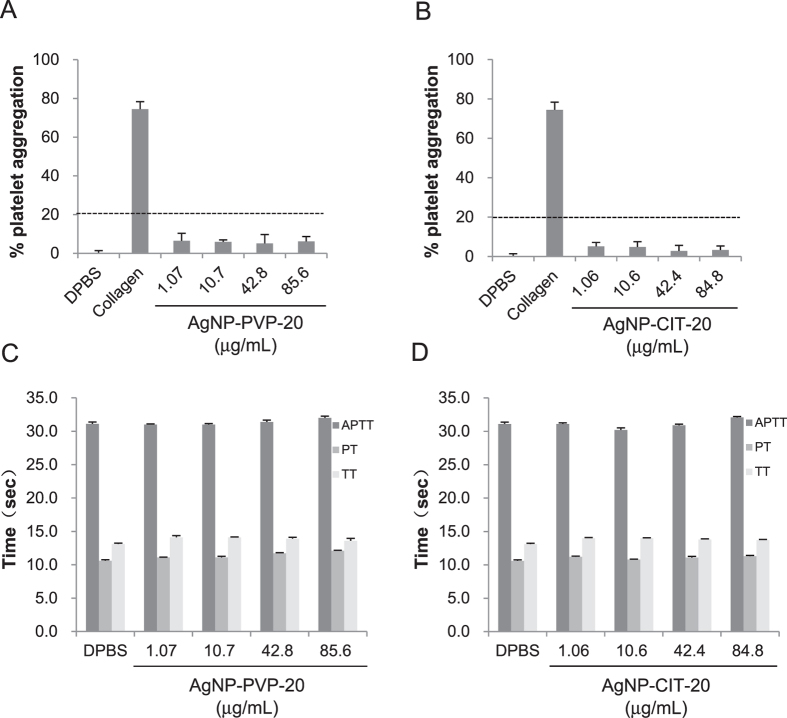
Effect of AgNPs on platelet aggregation and coagulation. Platelet aggregation was detected by incubating PRP with different concentrations of AgNP-PVP-20 (**A**) and AgNP-CIT-20 (**B**) for 15 min. 1 × DPBS and 1.0 mg/mL collagen were used as negative control and positive control, respectively. 20% of platelet aggregation was defined as the assay threshold (dash line). In the coagulation assay, APTT, PT and TT were separately tested after exposure PPP to AgNP-PVP-20 (**C**) and AgNP-CIT-20 (**D**) for 30 min.

To evaluate the effect of AgNPs on platelet aggregation, platelet count was determined after incubating platelet-rich plasma (PRP) with various concentrations of AgNPs for 15 min. As shown in Fig. 4, 1 mg/mL collagen as the positive control induced about 75% of platelet aggregation. Obviously, both AgNP-PVP-20 (Fig. 4A) and AgNP-CIT-20 (Fig. 4B) did not significantly accelerate platelet aggregation at the concentration from ~1 to ~80 μg/mL since their results did not exceed 20% as the assay threshold^31^. Furthermore, much higher concentrations of AgNPs up to ~500 μg/mL were also investigated for platelet aggregation (Fig. S2). There was still no apparent effect on the formation of platelet aggregation for both two kinds of AgNPs at the concentration of ~500 μg/mL. Moreover, as control, we checked whether the solvent citrate as well as Ag^+^ had a platelet aggregating activity or not. Fig. S2A,B showed that neither the corresponding concentrations of citrate nor Ag^+^ from 10 to 3000 ng/mL could give rise to platelet aggregation.

### Coagulation

Coagulation time for the three main pathways which are intrinsic, extrinsic and final common pathways can be evaluated by activated partial thromboplastin time (APTT), prothrombin time (PT) and thrombin time (TT), respectively. It has been known that the normal physiological levels for APTT, PT and TT were 25.1~36.5 s, 9.4~12.5 s, 10.3~16.6 s, respectively^26^,^27^. After treated with different concentrations of AgNP-PVP-20 (Fig. 4C) or AgNP-CIT-20 (Fig. 4D) for 30 min, platelet-poor plasma (PPP) was mixed by different reagents for testing clotting time. Our results showed that, from low concentration (about 1 μg/mL) to high concentration (about 500 μg/mL), AgNP-PVP-20 had no significant effect on plasma coagulation time for all three pathways (Fig. 4C and Fig. S2C). For AgNP-CIT-20, although it could not affect plasma coagulation at the dose of less than 212 μg/mL, it markedly prolonged APTT (about 70 s) by the intrinsic pathway at the high concentration of 530 μg/mL, which obviously exceeded the normal range. Moreover, we also quantified the influence of citrate and Ag^+^, which did not initiate any coagulation pathway as shown in Fig. S2C,D.

### Total complement activation function

**Figure 5:**
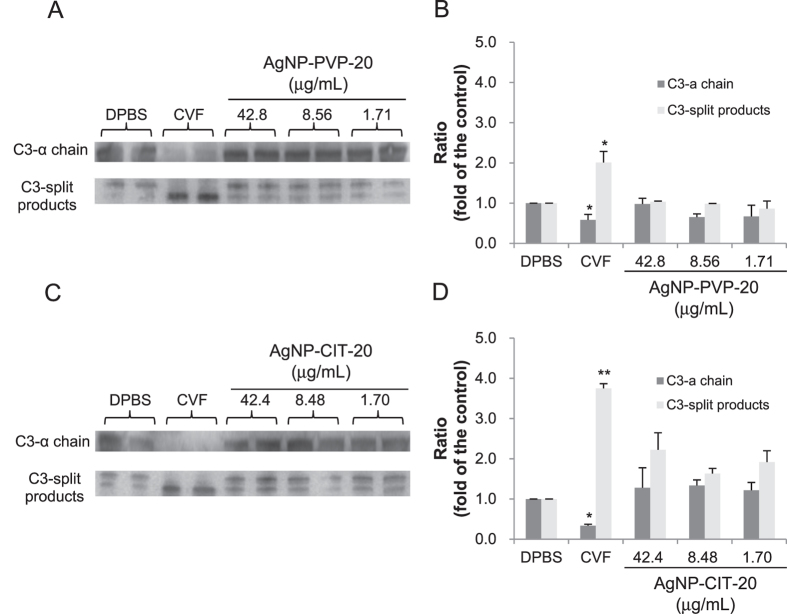
Qualitative analysis of total complement activation by Western blotting. The expression levels of C3-α chain and cleavage products were determined by Western blotting analysis after exposure to human normal plasma to indicated concentrations of AgNPs for 30 min at 37 °C. 1 × DPBS and CVF were used as negative control and positive control, respectively. Representative immunoblotting images (**A**,**C**) and the band integrated density analyzed by Image J (**B**,**D**) were presented. Ratio meant the intensity of each protein band relative to that of negative control group. *p < 0.05, **p < 0.01, significantly different from negative control. The gels were run under the same experimental conditions. The gels were copped from the full-length gels (Fig. S3).

Among several components (C1, C2…..C9) and factors (B, D, H, I, and P) in the complement system, C3 component, as the most abundant complement protein in serum, is cleaved following the activation of any of three main pathways^32^. According to different cleavage ways of C3, there are three products of about 40 kDa, which are C3α’, C3dg and C3d^33^. Therefore, we detected the cleavage of the majority of C3 component (C3α chain, 115 kDa) as well as the existence of C3 cleavage products by Western blotting. As shown in Fig. 5 (The full-length gels were presented in Supplementary Figure S3), cobra-venom factor (CVF) as positive control completely cleaved C3α chain as detected by the increased content of C3-split products compared to DPBS group as negative control. However, we found that after exposure to about 1 to 40 μg/mL AgNP-PVP-20 (Fig. 5A,B) or AgNP-CIT-20 (Fig. 5C,D) there was no remarkable change in the levels of C3α chain and C3-split products were not obviously increased. These results indicated that both AgNP-PVP-20 and AgNP-CIT-20 could not distinctly activate the complement system at the concentration range from about 1 to 40 μg/mL.

### Proteome analysis of blood plasma protein binding to AgNPs and predict biological process

**Figure 6:**
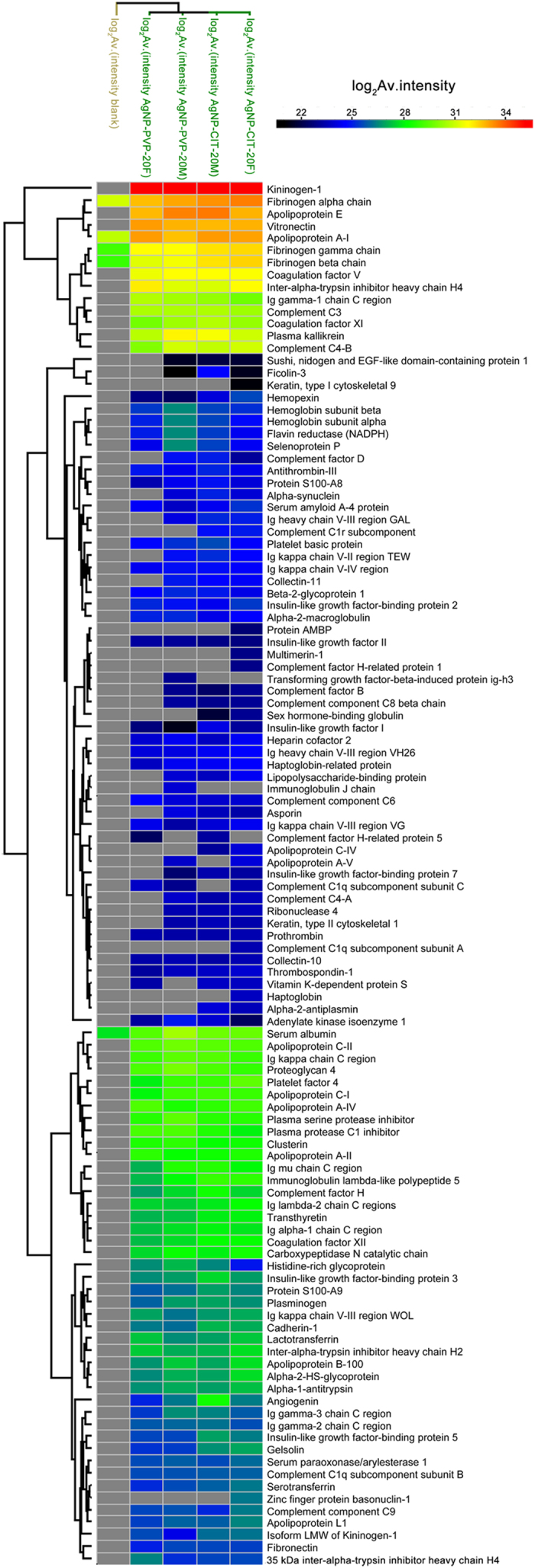
Heat map clusters of corona proteins identified on AgNPs illustrating sample similarities between genders as well as surface coatings.

In order to investigate plasma protein corona of AgNPs and how the composition of corona affected AgNPs blood compatibility, we analyzed the adsorbed protein on AgNP-PVP-20 and AgNP-CIT-20 using mass spectrometry (MS) based label free quantitative techniques (LFQ). In our experiments, AgNPs with corona were washed intensively to ensure the analysis was focused on hard protein corona which consists of proteins with high binding affinities^34^,^35^. As a result, the amounts of proteins identified were from 80 to 110 (Table S1, Fig. 6 and Fig. S4A). Moreover, our results are highly repeatable and have high correlations between biological repeats (Fig. S5). Meanwhile our results displayed over 70% corona proteins were shared and only few proteins with very low LFQ intensity were specific to female or male (Fig. 6 and Fig. S4A).

We further compared the proteins on corona with those in plasma for the difference in protein isoelectric point (pI), molecular weight (MW) and abundance. As shown in Fig. 6 and Fig. S4, 81 out of 113 proteins identified were present on all four AgNPs corona analysis. Fig. S6 showed that most identified proteins were abundant plasma proteins (concentrations varied from 10^−4^ to 1 mg/mL), while plasma proteins ranged from 10^−9^ to 10^1^ mg/mL. MW comparison revealed that more than 70% of corona proteins were less than 60 kDa, similar with the MW distribution of plasma proteins (Fig. S4B and S6). There was a slight preference of four samples (AgNP-PVP-20 and AgNP-CIT-20 with female and male plasma corona) consisting more proteins with 10–20 kDa and fewer proteins with 20–30 kDa when compared to plasma. pI analysis showed that more than 50% corona proteins had pI under 7 and percentage of proteins with pI between 4 and 5 were less than those in plasma (around 3% in corona compare to 10% in plasma), while proteins with pI from 6 to 7 (around 31% in corona compare to 24% in plasma) and from 8 to 9 (around 20% in corona compare to 12% in plasma) were enriched on the AgNPs corona (Fig. S4B and S7). When we compared the intensities of all identified corona proteins, we didn’t find any direct correlation as above between the intensities and the protein properties (Table S2). For example, although proteins with molecular weight of 10–20 kDa were enriched from plasma (Fig. S4), their intensities together were less than 3% of all corona proteins. This means that although more species of plasma proteins of 10–20 kDa are identified on AgNPs than others, they are not the majority of proteins on corona but still with low abundance.

**Figure 7:**
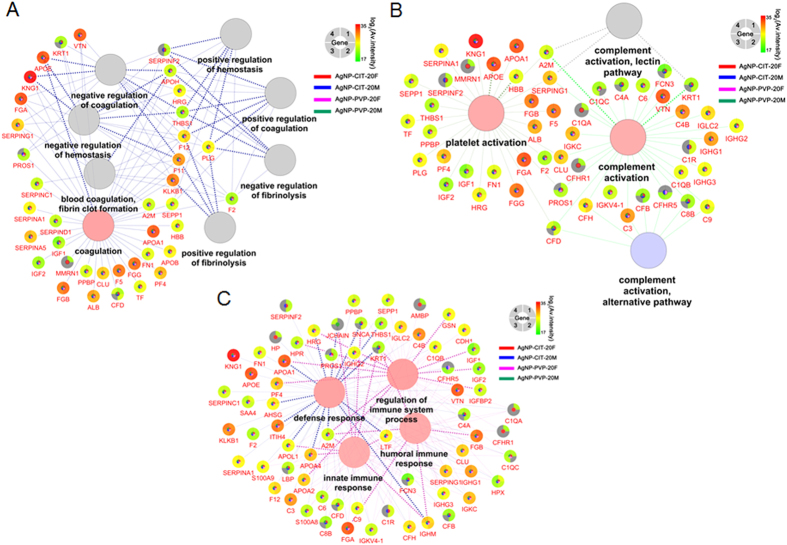
Proteins identified in each AgNPs corona by LC-MS were grouped by GO biological processes of coagulation, hemostasis (A), platelet or complement activation (B) and immune response (C). The relatively intensities of each protein (shown by gene name) were displayed in the gradient color on the outer layer of the circle for four experimental groups with green to red representing low to high intensity. Experimental groups with red for AgNP-CIT-20F, blue for AgNP-CIT-20M,magenta for AgNP-PVP-20F and green for AgNP-PVP-20M were shown in the inner layer with color filled when the protein is identified in that experiment. If a protein was not found in one group, the inner layer would not have the corresponding color of experimental group and its intensity in outer layer would fill with gray for that experiment.

To gain an insight into the mechanisms of AgNPs’ blood compatibility, we applied Gene Ontology (GO) analysis to see what biological processes that identified corona proteins were involved (Table S3). Figure 7 showed GO biological processes involving coagulation, hemostasis, platelet or complement activation and immune responses along with relevant proteins (shown in gene name) found on coronas. Notably, corona proteins contained both promoting and counteracting effects for these processes. Moreover, our quantitative data also provided the relative intensity which can estimate the approximate abundance of corona proteins on AgNPs. According to the intensity distribution (Fig. 6 and Table S1), the major absorbed proteins and low-abundant proteins on coronas varied from 2^21^ to 2^35^ which had a difference of five magnitudes. Estimated from LFQ intensity (Table 2 and Table S1), kininogen-1(HMWK) was the dominating protein binding to the surface of 20 nm AgNPs, occupying 36–42% of all bounded proteins. Other abundant proteins were apolipoprotein E (6–10%), apolipoprotein A1 (5–8%), fibrinogen alpha chain (6–9%) and vitronectin (5–8%).

## Discussion

Because of their broad antimicrobial spectrum as well as greater efficacy against some bacteria, AgNPs have been widely used in consumer products than any other nanomaterials, especially in both consumer and biomedical applications^5^,^6^,^36^. Despite the global use of AgNPs products, more detailed information concerning their biological effects are still required. Therefore, in this study, we have systematically investigated hemolysis, platelet aggregation, coagulation, lymphocyte proliferation, the activation of complement system and the characterization of plasma protein coronas after the treatment with AgNP-PVP-20 and AgNP-CIT-20. Therefore, through combining our experimental data with proteomic analysis, we can more deeply comprehend the blood compatibility of AgNPs.

As mentioned in our study, both 20 nm AgNPs could arouse dose-dependent hemolysis. It has been found that AgNPs have hemolytic effects by changing membrane integrity and surface characteristics, in which the hemolytic effect is not only size-dependent^19^,^37^. Kwon *et al.* reported an appropriate size and dosage of AgNPs could alleviate human erythrocyte hemolysis response^37^. AgNPs might cause pore formation on the membrane of erythrocytes and ultimately result in osmotic lysis^37^. However, it is not yet clear about the exact reason for erythrocyte membrane damage. Low concentrations of Ag ion (Ag^+^) could induce hemolysis and RBC death *in vitro*, which is possibly associated with reactive oxygen species (ROS)^37^,^38^. In addition, lipid peroxidation (LPO) as the oxidative deterioration of cell membrane lipids is also supposed to contribute to hemolysis^39^. Thus, although the mechanism explaining AgNPs-triggered hemolysis has not yet been completed understood, we speculated that the mechanism of disrupting erythrocytes by 20 nm AgNPs coated with PVP and citrate tested in our study might be correlated with Ag^+^ release and oxidative stress.

For lymphocyte proliferation, our results showed that at the concentration of 10–40 μg/mL, AgNP-PVP-20 and AgNP-CIT-20 were quite cytotoxic to human lymphocytes although at the low concentration of about 1 μg/mL, both AgNPs could slightly promote lymphocyte proliferation. Then, we also found that the cytotoxic pattern of both AgNP-CIT-20 and AgNP-PVP-20 were comparable with that of Ag^+^ (Fig. S1B). Hence, it was suspected that the lymphocyte toxicity of both AgNPs in our experiments were primarily due to Ag^+^ release^40^. Studies on mechanism of Ag^+^ toxicity have been reported. Ag^+^ seems to be able to cause mitochondria perturbation through interactions with thiol groups of mitochondrial inner membrane and further disrupt mitochondria functions^41^. Metal ion including Ag^+^ may also alter metabolic activities during long-term exposure^42^. Naturally, besides Ag^+^ dissolution, there might be additional factors correlated with the lymphocyte toxicity of AgNPs, including nanoparticles (NPs) accumulation in cells, subsequent cellular uptake, intracellular transport, and storage as well as DNA damage in the nucleus^40^,^43^,^44^. In addition, many papers have reported that both AgNPs and Ag^+^ could induce cytotoxicity through an increase in oxidative stress^43^,^45^,^46^. So we supposed the production of ROS (reactive oxygen species) might be the main cause for lymphocyte toxicity by AgNPs. A recent paper also revealed the epigenetic mechanism of AgNPs and Ag^+^ by integrated mRNA and microRNA profiling^47^. In addition, the down-regulation of ERK pathway may be involved in the cytotoxicity of AgNPs in the human leukemic T cell line Jurkat^48^. However, there are still other studies showing that either the spontaneous proliferation of T-cells or the proliferative response to stimuli was not notably affected by the presence of AgNPs *in vitro* or *in vivo*^44^,^49^,^50^. And for our case, we need to carry on more study to confirm the detailed mechanism in the future work.

Although it has been reported about the innate antiplatelet properties of AgNPs^21^,^51^,^52^ and AgNPs-induced platelet aggregation^18^, our result indeed suggested that platelet aggregation could not be obviously observed after treatment with 20 nm AgNPs coated with PVP or citrate at the concentrations from 1 to 500 μg/mL, which was similar to the data of AgNPs between 10–15 nm^19^. For *in vivo* experiment, both negative and positive effects on platelet aggregation are also observed for AgNPs^18^,^21^,^53^. Our observation that AgNP-CIT-20 at a higher concentration had anticoagulant effect on the intrinsic pathway in blood, which is consistent with reported work on 24 nm 67 μg/mL AgNPs^24^. However, the opposite result has been reported that 15 nm 30 μg/mL AgNPs can bring an elevated risk of thrombosis by the activation of intrinsic coagulation cascade^19^. Thereby, the reason for the discrepancy and the mechanisms of AgNPs effects on platelet aggregation and coagulation require more additional studies, which may arise from different physicochemical properties of the used AgNPs samples, such as size, surface coatings, surface charge and so on.

Owing to their high surface free energy, NPs can adsorb proteins to form corona in biological fluids and the modified surface consequently affect the bioactivity and toxicity of NPs^54^,^55^. In our experiments, the amounts of proteins identified is consistent with previous studies by others suggesting that the hard protein corona typically contains less than 200 species of proteins^56^,^57^,^58^,^59^. It has been shown that some proteins levels, such as monocyte chemotactic protein-1 (MCP1) and high sensitivity C-reactive protein are different in female and male plasma^60^,^61^. We thus examined the corona from female and male plasma separately. As our results have showed, only few low LFQ intensity proteins were specific to female or male, such as transforming growth factor-beta-induced protein ig-h3 (TGFBI), immunoglobulin J-chain and zinc finger protein bosonuclin-1. However these proteins haven’t been reported to specific to male or female plasma, but rather results from few amount of non-specific binding of AgNPs or stochastic character of mass spectrometry analysis. Therefore, our result revealed that for these two materials the gender may not be a major factor affecting corona composition.

Further analysis for corona protein’s molecular weight (MW), isoelectric point (pI) and abundance indicate the corona composition of AgNP-CIT-20 and AgNP-PVP-20 consist of low molecular weight proteins (<60 kDa), which is different from previous report of the MW distribution of protein corona for 30 nm silica NPs contains above 40% high molecular weight proteins (>150 kDa)^58^. pI analysis showed that more than 50% corona proteins had pI under 7, which may be due to the fact that most of plasma proteins were acidic. We found that percentage of proteins with pI between 4 and 5 were less than those in plasma (around 3% in corona compare to 10% in plasma), while proteins with pI from 6 to 7 (around 31% in corona compare to 24% in plasma) and from 8 to 9 (around 20% in corona compare to 12% in plasma) were enriched on the AgNPs corona (Fig. S4B and S7). We proposed that the enrichment of proteins with relatively high pI on these nanoparticles was due to the negative charge of the surface modification. This indicated that the interaction between plasma proteins and AgNPs was not random. These comparisons were made using the proteins species identified on corona and plasma proteins with concentration in Plasma Proteome Database^62^. When we compared the intensities of all identified corona proteins, we didn’t find any direct correlation as above between the intensities and the protein properties (Table S2). For example, although proteins with molecular weight of 10–20 kDa were enriched from plasma (Fig. S4), their intensities together were less than 3% of all corona proteins. This means that although more species of plasma proteins of 10–20 kDa are identified on AgNPs than others, they are not the majority of proteins on corona but still with low abundance. Many factors can affect corona composition, which include sizes, modifications and cores of nanoparticles as well as MW, pI, abundance, protein interactions and even structure properties of plasma proteins. Therefore, a lot of work will be required to study the mechanism of corona formation and protein selectivity, which may provide a comprehensive understanding of the biological effects AgNPs in the blood.

Early studies have demonstrated that proteins binding onto NPs are among the key factors affecting the biocompatibility^29^,^63^. Certain protein components, such as apolipoproteins^63^ and opsonins^64^ on a corona may enhance the uptake of NPs by endothelial cells. The enrichment of complement C3 will modulate complement activation^28^. Besides, in the blood system, many biological processes are triggered by cascade reactions of plasma proteins^65^,^66^. To gain an insight into the mechanisms of AgNPs’ blood compatibility, we applied Gene Ontology (GO) analysis to see what biological processes that identified corona proteins were involved (Table S3). Fig. 7 showed GO biological processes involving coagulation, hemostasis, platelet or complement activation and immune responses along with relevant proteins (shown in gene name) found on coronas. Notably, corona proteins contained both promoting and counteracting effects for these processes. Moreover, our quantitative data also provided the relative intensity which can estimate the approximate abundance of corona proteins on AgNPs. According to the intensity distribution (Fig. 6 and Table S1), the major absorbed proteins and low-abundant proteins on coronas varied from 221 to 235 which had a difference of five magnitudes. Estimated from LFQ intensity (Table 2 and Table S1), kininogen-1(HMWK) was the dominating protein binding to the surface of 20 nm AgNPs, occupying 36–42% of all bounded proteins. This was consistent with the study of citric modified AgNPs corona in serum by Elechiguerra JL *et al.*^7^. Other abundant proteins were apolipoprotein E (6–10%), apolipoprotein A1 (5–8%), fibrinogen alpha chain (6–9%) and vitronectin (5–8%).

Among the leading abundant corona proteins, HMWK, which has no activity per se is a cofactor for the activation of plasma kallikrein, factor XI and factor XII in the “contact-kinin system”^67^,^68^. However, recent studies implied that despite their initial description as initiators of contact-kinin system, these proteins have anti-coagulant and pro-fibrinolytic properties. In particular, HMWK can inhibit platelet aggregation and have anti-thrombotic functions in a direct or indirect way^69^,^70^,^71^. In addition, another functional family of plasma proteins on AgNPs corona was apolipoproteins participating in the transport of lipid and cholesterol (Table 2 and Fig. 6). Apolipoprotein E and A1 are also found to be significantly enriched on silica NPs^58^, which enhance the uptake of NPs by endothelial cells^58^,^63^. Apolipoprotein E and A1 both display anti-inflammatory properties by modulating innate immune responses^72^, and inhibit platelet aggregation through the L-arginine-nitric oxide pathway^73^,^74^. Thus, the abundant interacted proteins listed by GO processes in Fig. 7 were mostly negative regulators of platelet aggregation and thrombosis. Our above experimental results showed that both AgNP-PVP-20 and AgNP-CIT-20 did not significantly accelerate platelet aggregation and plasma coagulation. This was possibly related with the observation that there were more negative regulators than positive ones involving platelet aggregation and thrombosis processes on corona. The complement system in humans includes about 35–40 proteins in the blood plasma and on the surface of cells^65^ and the complement protein C3 (C3) plays a central role in the classical, alternative and lectin activation pathways of the complement system. By analyzing NPs corona, Yu *et al.* found C3 was a major absorbate on glycopolymer-grafted NPs (14–19%) and can modulate and amplify the complement activation^28^. However, in our result, C3 occupies only 1.1–1.4% among all AgNPs bound proteins. Indeed, C3 was not enriched on AgNP-PVP-20 and AgNP-CIT-20 as in plasma C3 is about 2% of total blood plasma protein^65^. It is generally believed that proteins were more likely to trigger their biological responses when in higher abundance. Our finding about few C3 on AgNPs, together with more negative than positive factors involving platelet aggregation and thrombosis might be responsible for the observations that AgNP-PVP-20 and AgNP-CIT-20 had no significant effect on plasma coagulation, platelet aggregation and complement activation. Nevertheless, all these biological processes are much complicated, thus more experiments will be required to reveal and validate the effects of corona composition on blood biocompatibility.

## Conclusions

In summary, we have mapped the potent biological effects of AgNPs on blood. For RBCs, AgNP-PVP-20 at ~40 μg/mL could cause hemolysis to 19%. Both AgNP-PVP-20 and AgNP-CIT-20 were quite toxic to lymphocytes since they severely inhibited both lymphocyte proliferation and viability. AgNPs did not show any obvious effect on platelet aggregation, coagulation cascade and the activation of complement system at less than 40 μg/mL. However, it’s worth noting that at very high concentration of about 500 μg/mL, AgNP-CIT-20 markedly inhibited the coagulation process. These findings provided strong evidence that the bio-effects induced by AgNPs in blood were dose-dependent. The data from proteomic analysis of protein coronas indicated that citrate or PVP coated AgNPs preferred to recruit negative regulators or at least not stimulator of platelet aggregation and coagulation factors in plasma. It supported our experimental data of bio-effects analysis of AgNPs in blood. Our results also suggest that when we predict biological functions of NPs, besides the protein species (IDs), it is as important to quantify the amount of corona proteins. Taken together, our observations provide a systematic overview of AgNPs blood biocompatibility.

## Methods

All experiments were performed in accordance with relevant guidelines and regulations and approved by the institutional committee (National Center for Nanoscience and Technology, P.R.China). The authors declare that the informed consent was obtained from all subjects.

### Characterization of AgNPs

Endotoxin-free AgNPs of 20 nm coated with polyvinyl pyrrolidone (PVP, AgNP-PVP-20) and with citrate (AgNP-CIT-20) were from NanoComposix USA (Catalog No. AGPB20 and AGCB20, respectively). The morphology and sizes of AgNPs at 10 μg/mL were imaged by transmission electron microscopy (TEM, Tecnai F20, FEI, USA). The ultraviolet-visible (UV-vis) absorption spectra of AgNPs were recorded by Infinite^®^ M200 microplate reader (Tecan, Switzerland) from 300 to 600 nm. The zeta potential and hydrodynamic size of AgNPs were measured by a Malvern Zetasizer Nano ZS (Malvern Instruments, Worcestershire, UK). AgNPs were dispersed in Ca^2+^/Mg^2+^ free Dulbecco’s Phosphate-Buffered Saline (DPBS, Cellgro, USA), RPMI-1640 culture medium (Cellgro, USA) with 10% fetal bovine serum (FBS, HyClone, USA) before measurement, respectively. For AgNPs with plasma, AgNPs were mixed with human plasma at 1:3 (v/v) and incubated for 10 min.

### Human blood samples

All human blood samples in this manuscript were from healthy volunteers and used with institutional bioethics approval.

### Hemolysis assay

The hemolysis assay was carried out according to the protocol from National Cancer Institute (NCI)^16^,^75^. Briefly, the whole blood was required by the calculated plasma free hemoglobin (PFH) concentration below 1.0 mg/mL. Pooled blood obtained from at least three healthy donors was diluted with 1 × DPBS to adjust total blood hemoglobin (TBH) concentration to 10 ± 2 mg/mL (TBHd). 100 μL 1 × DPBS as blank, AgNPs solution in 1 × DPBS, polyethylene glycol (PEG, Sigma-Aldrich, USA) as negative control or Triton-X-100 (Sigma-Aldrich, USA) as positive control were added to 700 μL 1 × DPBS in different tubes. Then, 100 μL of TBHd was added followed by incubating at 37 °C for 3 h ± 15 min. Then, the mixture was centrifuged at 800× *g* for 15 min. 100 μL supernatant and 100 μL cyanmethemoglobin (CMH) reagent were added to a 96-well plate. The CMH reagent was prepared by mixing 1000 mL Drabkin’s reagent and 0.5 mL of 30% Brij 35 solution (Sigma-Aldrich, USA). The absorbance (OD) at 540 nm was determined by the Infinite^®^ M200 microplate reader (Tecan, Switzerland). The percentage of hemolysis was calculated by the equation: % hemolysis = ((HC_sample_ − HC_blank control_)/(HC_TBHd_ − HC_blank control_)) × 100%. For each term of the equation, the OD value was already subtracted by their background interference.

### The proliferation assay of peripheral lymphocytes

Lymphocyte proliferation was measured using CellTiter 96^®^ AQueous One Solution Reagent (Promega, USA). Peripheral blood mononuclear cells (PBMCs) were collected from fresh human whole blood by Ficoll solution (GE Healthcare, USA). After washing cells by 1 × DPBS, 1 × 10^5^ PBMCs in 100 μL RPMI-1640 medium were seeded in each well of 96-well round bottom plates. Then AgNPs, RPMI-1640 as negative control, 10 μg/mL phytohemagglutinin (PHA-M, Sigma-Aldrich, USA) as positive control, blank control were added into the plate, followed by incubating at 37 °C for three days. 40 μL MTS reagent was added into each well. After 4 h incubation at 37 °C, the absorbance (OD) at 490 nm as well as 650 nm as a reference wavelength was detected via an EnSpire^®^ multimode plate reader (PerkinElmer, USA). The percentage of cell proliferation was calculated as the following formula: % cell proliferation = (Mean OD_sample_ - Mean OD_negative control_)/Mean OD_negative control_ × 100%.

### Platelet aggregation assay

Platelet-rich plasma (PRP) was obtained by pooling fresh human plasma from at least three donors by centrifugation at 200 × *g* for 8 min. 25 μL of AgNPs solution diluted by 1 × DPBS, 1 × DPBS as negative control or 1.0 mg/mL collagen (Sigma-Aldrich, USA) as positive control were mixed with 100 μL PRP, respectively. Then, all samples were incubated at 37 °C for 15 min. The platelet counting (PC) was performed by XS-800i automatic blood analyzer (Sysmex, Japan). The percentage of platelet aggregation (% platelet aggregation) was calculated by ((PC_negative control_ − PC_sample_)/PC_negative control_) × 100%.

### Coagulation assay

Platelet-poor plasma (PPP) was collected through the centrifugation of fresh human whole blood from at least three donors at 2,500× *g* for 10 min and pooled. 50 μL AgNPs solution diluted by 1 × DPBS and 1 × DPBS as negative control were mixed with 500 μL of PPP. All samples were incubated at 37 °C for 30 min, and then analyzed by an ACT-TOP automatic coagulation analyzer (Instrumentation Laboratory. Co, USA) for prothrombin time (PT), activated partial thromboplastin time (APTT), and thrombin time (TT).

### Total complement activation

The qualitative analysis of total complement activation was performed by referring to the NCI protocol^76^. Equal volumes (10 μL) of veronal buffer, pooled human plasma and a test-sample (AgNPs in 1 × DPBS, 1 × DPBS as negative control, cobra venom factor (CVF) as positive control) were combined in a microcentrifuge tube. After briefly mixed, the tubes were incubated for 30 min at 37 °C. Subsequently, the cleavage products (about 43 kDa) of the C3-α chain (approximately 115 kDa) were investigated by the anti-C3 antibody (EMD Biosciences, Calbiochem, USA) by sodium dodecyl sulfate-polyacrylamide gel electrophoresis (SDS-PAGE). The immunoblotting image was evaluated by integrated density (area × mean gray value) using NIH Image J.

### Plasma protein binding

Human plasma was obtained from 15 healthy males and females aged 25–45 years and pooled. 1 mg/mL 20 nm AgNPs were incubated for 3 h at room temperature with 3 volumes plasma to ensure the ratio of plasma volume to particle surface more than 5.55 mL/m^2 ^77. The samples were loaded onto a sucrose cushion (0.5 M in PBS) and centrifuged for 20 min at 19,000× *g*, 4 °C. Pellets were washed with 1 × PBS for three times and 0.1 × PBS for another three times. The 2 mM citric buffer as blank control was treated with the same procedure as AgNPs.

### Protein extraction and in-solution digestion

Proteins on nanoparticles were eluted by lysis buffer (6 M guanidine hydrochloride, 50 mM Tris, 20 mM TCEP, 50 mM IAA), followed by sonication at 4 °C for 1 min and incubation in dark for 40 min. The lysates were boiled for 5 min and nanoparticles were removed by centrifugation. In-solution two-step digestion procedure were performed as described^78^. Briefly, the lysates were treated by Lys-C (Promega Corporation, Madison, USA) at a ratio of 1:100 (micrograms of enzyme to micrograms of protein) for 4 h at 37 °C, and the digestion mixture was diluted with 25 mM NH_4_HCO_3_. Then samples were incubated with trypsin (Promega Corporation, Madison, USA) overnight at 37 °C. Finally, peptides were acidified and desalted by C18 (3 M, USA)^79^.

### Label free quantification

Approximately 0.5–1 μg of peptides were loaded for 80 min gradients, respectively. Peptides were separated on an 20 cm reversed phase capillary emitter column (inner diameter 100 μm, 5 μm Venusil XBP C18 resin (Agela Technologies, China)) and analyzed on the Q Exactive instrument (Thermo Fisher Scientific). Peptides were loaded in 0.2% (v/v) formic acid solution and eluted with a nonlinear 80 min gradient of 5–30% buffer B (0.1% (v/v) formic acid, 90% (v/v) acetonitrile) at a flow rate of 300 nL/min. MS data were acquired by Thermo X calibur (2.0) with an data-dependent MS/MS scans (TopN = 15). Target value for the full MS scan was 3 × 10^6^ in the 320–1,700 m/z range with a maximum injection time of 20 ms and a resolution of 70,000 at m/z of 200. Isolation window was 1.5 m/z and normalized collision energy of 27. MS/MS scans resolution was 17,500 at m/z 200 with an ion target value of 1 × 10^5^ and a maximum injection time of 60 ms. To avoid peptide’s repeated sequencing, exclusion time was set to 60 s.

MS raw files were processed with MaxQuant software (version 1.4.1.2) with label free quantification work flow (MaxLFQ)^80^. The peak lists were searched against the human Uniprot FASTA (version 20 April 2014 (88725 entries)) with reversed protein sequences and a common contaminants database (247 entries) by Andromeda search engine^81^. The search included cysteine carbamidomethylation and variable modifications of methionine oxidation and protein N-terminal acetylation. Enzyme specificity was set as C-terminal to arginine and lysine, and a maximum of two missed cleavages were allowed in the database search. Peptides with at least six amino acids were considered for identification. The false discovery rate for both peptides and proteins was set at 1%.

### Bioinformatics analysis

Data analysis was performed with Perseus software in the MaxQuant computational platform and by R statistical computing environment^82^. The three technological repeats of each sample were set as one experiment when running MaxLFQ process, and the average value (Av.) of biological repetition of each identified protein’s LFQ intensity was used in following analysis. Gene ontology (GO) biological process of proteins and their interactions network were performed by Cytoscape^83^ plugged ClueGO + CluePedia^84^ with *P*-values (Bonferroni step down correction) <0.05^85^. The concentrations of plasma proteins were derived from Plasma Proteome Database (PPD; http://www.plasmaproteomedatabase.org/)^62^. For correlation analysis of corona proteins’ pI and MW with their LFQ intensity, two tailed Spearman test was used.

### Statistical analysis

Statistical analysis was performed by Student’s t-test. All values were expressed as mean ± standard deviation (SD) of duplicate or triplicate samples in a representative experiment. All experiments were done independently at least three times. A *P*-value less than 0.05 was considered as statistical significance.

## Additional Information

**How to cite this article**: Huang, H. *et al.* An Evaluation of Blood Compatibility of Silver Nanoparticles. *Sci. Rep.* **6**, 25518; doi: 10.1038/srep25518 (2016).

## Supplementary Material

Supplementary Information

## Figures and Tables

**Table 1:** Characterization of AgNPs*.

Characterization	AgNP-PVP-20	AgNP-CIT-20
Size/nm (TEM)	21.6 ± 4.8	24.3 ± 4.5
Morphology	spherical	spherical
Λmax/nm (Water)	395	395
Λmax/nm (Plasma)	400	405
d_H_/nm (Water)	58.6 ± 2.4	26.6 ± 1.89
ζ/mV (Water)	−25.1 ± 3.7	−37.0 ± 0.4
d_H_/nm (1 × DPBS)	99.5 ± 2.7	654.7 ± 5.1
ζ/mV (1 × DPBS)	−6.4 ± 0.7	−16.1 ± 0.8
d_H_/nm (RPMI-1640)	93.5 ± 1.2	115.3 ± 0.7
ζ/mV (RPMI-1640)	−6.6 ± 0.3	−9.9 ± 0.2
d_H_/nm (Plasma)	53.3 ± 2.6	58.1 ± 1.6
ζ/mV (Plasma)	−21.2 ± 0.6	−26.1 ± 0.2

^*^The data are expressed as mean ± SD of duplicate experiments.

**Table 2:** Top 20 corona proteins identified after incubating with plasma for 3 h.

No.	AgNP-CIT-20F		AgNP-CIT-20M		AgNP-PVP-20F		AgNP-PVP-20M	
	Protein(gene)	Abundance*	Protein(gene)	Abundance*	Protein(gene)	Abundance*	Protein(gene)	Abundance*
1	Kininogen-1 (KNG1)	35.42165	Kininogen-1 (KNG1)	35.57028333	Kininogen-1 (KNG1)	35.43163333	Kininogen-1 (KNG1)	35.25791667
2	Fibrinogen alpha chain (FGA)	33.46088334	Apolipoprotein E (APOE)	33.55425	Apolipoprotein A-I (APOA1)	33.09685	Apolipoprotein E (APOE)	33.43363334
3	Apolipoprotein A-I (APOA1)	33.00553334	Fibrinogen alpha chain (FGA)	33.23846667	Vitronectin (VTN)	33.09233334	Fibrinogen alpha chain (FGA)	32.95638334
4	Vitronectin (VTN)	32.81335	Apolipoprotein A-I (APOA1)	33.12035	Apolipoprotein E (APOE)	32.71623334	Vitronectin (VTN)	32.7894
5	Apolipoprotein E (APOE)	32.80266667	Vitronectin (VTN)	32.71365	Fibrinogen alpha chain (FGA)	32.6453	Apolipoprotein A-I (APOA1)	32.60831667
6	Fibrinogen beta chain (FGB)	32.36591667	Fibrinogen gamma chain (FGG)	32.19898334	Inter-alpha-trypsin inhibitor heavy chain H4 (ITIH4)	32.06611667	Plasma kallikrein (KLKB1)	31.95661667
7	Fibrinogen gamma chain (FGG)	32.30948334	Fibrinogen beta chain (FGB)	32.17975	Fibrinogen gamma chain (FGG)	31.78608333	Coagulation factor V (F5)	31.73656667
8	Inter-alpha-trypsin inhibitor heavy chain H4 (ITIH4)	31.84591667	Coagulation factor V (F5)	31.79088334	Coagulation factor V (F5)	31.4815	Fibrinogen gamma chain (FGG)	31.70983334
9	Coagulation factor V (F5)	31.69698333	Plasma kallikrein (KLKB1)	31.64233333	Fibrinogen beta chain (FGB)	31.29676667	Fibrinogen beta chain (FGB)	31.59158334
10	Complement C4-B (C4B)	31.10568334	Inter-alpha-trypsin inhibitor heavy chain H4(ITIH4)	31.1836	Plasma kallikrein (KLKB1)	30.7676	Inter-alpha-trypsin inhibitor heavy chain H4 (ITIH4)	31.2543
11	Plasma kallikrein (KLKB1)	31.05406667	Complement C4-B(C4B)	30.88695	Ig gamma-1 chain C region (IGHG1)	30.66883334	Complement C4-B (C4B)	31.11485
12	Complement C3 (C3)	30.40113333	Coagulation factor XI(F11)	30.71216667	Complement C3 (C3)	30.58146667	Complement C3 (C3)	30.61288334
13	Coagulation factor XI (F11)	30.27568334	Complement C3(C3)	30.61853334	Complement C4-B (C4B)	30.01456667	Serum albumin (ALB)	30.45698334
14	Ig gamma-1 chain C region (IGHG1)	29.76771667	Ig gamma-1 chain C region(IGHG1)	30.25201667	Coagulation factor XI (F11)	29.82581667	Ig gamma-1 chain C region (IGHG1)	30.44638334
15	Serum albumin (ALB)	29.64955	Serum albumin(ALB)	29.70115	Serum albumin (ALB)	29.39855	Coagulation factor XI (F11)	30.38823334
16	Platelet factor 4 (PF4)	29.5006	Apolipoprotein C-II (APOC2)	29.44895	Apolipoprotein C-II (APOC2)	29.33451667	Proteoglycan 4 (PRG4)	29.84238334
17	Apolipoprotein C-II (APOC2)	29.30501667	Ig kappa chain C region (IGKC)	29.35978334	Apolipoprotein A-IV (APOA4)	29.31788334	Apolipoprotein C-II (APOC2)	29.7589
18	Apolipoprotein A-IV (APOA4)	29.1102	Proteoglycan 4 (PRG4)	29.27093334	Proteoglycan 4 (PRG4)	29.2332	Plasma serine protease inhibitor (SERPINA5)	29.48916667
19	Ig kappa chain C region (IGKC)	28.98816667	Apolipoprotein C-I (APOC1)	29.11438334	Plasma protease C1 inhibitor (SERPING1)	29.203	Ig kappa chain C region (IGKC)	29.47166667
20	Apolipoprotein C-I (APOC1)	28.983	Platelet factor 4 (PF4)	28.99733334	Ig kappa chain C region (IGKC)	29.03638334	Plasma protease C1 inhibitor (SERPING1)	29.26511667

^*^Represent the log2 (Av.intensity) of protein identified with LFQ.

## References

[b1] Guidance for industrial minerals on the implementation of the European Commission recommendation of 18 October 2011 on the definition of nanomaterial (2011/696/EU). *The European Commission* L275/38, 1–27 (2011).

[b2] Leucuta S. E. Nanotechnology for delivery of drugs and biomedical applications. Curr. Clin. Pharmacol. 5, 257–280 (2010). 20925643 10.2174/157488410793352003

[b3] Chaloupka K., Malam Y. & Seifalian A. M. Nanosilver as a new generation of nanoproduct in biomedical applications. Trends Biotechnol. 28, 580–588 (2010). 20724010 10.1016/j.tibtech.2010.07.006

[b4] Lansdown A. B. A pharmacological and toxicological profile of silver as an antimicrobial agent in medical devices. Adv. Pharmacol. Sci. 2010, 910686; 10.1155/2010/910686 (2010). 21188244 PMC3003978

[b5] Ahamed M., Alsalhi M. S. & Siddiqui M. K. Silver nanoparticle applications and human health. Clin. Chim. Acta 411, 1841–1848 (2010). 20719239 10.1016/j.cca.2010.08.016

[b6] Beer C., Foldbjerg R., Hayashi Y., Sutherland D. S. & Autrup H. Toxicity of silver nanoparticles - nanoparticle or silver ion? Toxicol. Lett. 208, 286–292 (2012). 22101214 10.1016/j.toxlet.2011.11.002

[b7] Elechiguerra J. L. *et al.* Interaction of silver nanoparticles with HIV-1. J. Nanobiotech. 3, 6 10.1186/1477-3155-3-6 (2005). PMC1190212 15987516

[b8] Tian J. *et al.* Topical delivery of silver nanoparticles promotes wound healing. ChemMedChem 2, 129–136 (2007). 17075952 10.1002/cmdc.200600171

[b9] Wong K. K. *et al.* Further evidence of the anti-inflammatory effects of silver nanoparticles. ChemMedChem 4, 1129–1135 (2009). 19405063 10.1002/cmdc.200900049

[b10] Kalishwaralal K., BarathManiKanth S., Pandian S. R. K., Deepak V. & Gurunathan S. Silver nanoparticles impede the biofilm formation by *Pseudomonas aeruginosa* and *Staphylococcus epidermidis*. Colloid Surface B 79, 340–344 (2010). 10.1016/j.colsurfb.2010.04.014 20493674

[b11] Chen X. & Schluesener H. J. Nanosilver: A nanoproduct in medical application. Toxicol. Lett. 176, 1–12 (2008). 18022772 10.1016/j.toxlet.2007.10.004

[b12] Sung J. H. *et al.* Subchronic inhalation toxicity of silver nanoparticles. Toxicol. Sci. 108, 452–461 (2009). 19033393 10.1093/toxsci/kfn246

[b13] Laloy J. *et al.* Impact of silver nanoparticles on haemolysis, platelet function and coagulation. Nanobiomedicine 1, 10.5772/59346 (2014). PMC6029236 30023015

[b14] EN BS ISO 10993-4:2009 Biological evaluation of medical devices. Part 4: Selection of tests for interactions with blood. 10.3403/30194461U (2010).

[b15] Arzoumanian L. What is hemolysis, what are the causes, what are the effects? BD Tech Talk 2, 1–3 (2003).

[b16] Dobrovoiskaia M. A. *et al.* Method for analysis of nanoparticle hemolytic properties *in vitro*. Nano Lett. 8, 2180–2187 (2008). 18605701 10.1021/nl0805615 PMC2613576

[b17] Delvaeye M. & Conway E. M. Coagulation and innate immune responses: can we view them separately? Blood 114, 2367–2374 (2009). 19584396 10.1182/blood-2009-05-199208

[b18] Jun E. A. *et al.* Silver nanoparticles enhance thrombus formation through increased platelet aggregation and procoagulant activity. Nanotoxicology 5, 157–167 (2011). 20822370 10.3109/17435390.2010.506250

[b19] Krajewski S. *et al.* Hemocompatibility evaluation of different silver nanoparticle concentrations employing a modified Chandler-loop *in vitro* assay on human blood. Acta Biomater. 9, 7460–7468 (2013). 23523936 10.1016/j.actbio.2013.03.016

[b20] Choi J., Reipa V., Hitchins V. M., Goering P. L. & Malinauskas R. A. Physicochemical characterization and *in vitro* hemolysis evaluation of silver nanoparticles. Toxicol. Sci. 123, 133–143 (2011). 21652737 10.1093/toxsci/kfr149

[b21] Shrivastava S. *et al.* Characterization of antiplatelet properties of silver nanoparticles. ACS Nano 3, 1357–1364 (2009). 19545167 10.1021/nn900277t

[b22] Stevens K. N. J. *et al.* The relationship between the antimicrobial effect of catheter coatings containing silver nanoparticles and the coagulation of contacting blood. Biomaterials 30, 3682–3690 (2009). 19394689 10.1016/j.biomaterials.2009.03.054

[b23] Shrivastava S. *et al.* Negative regulation of fibrin polymerization and clot formation by nanoparticles of silver. Colloid Surface B 82, 241–246 (2011). 10.1016/j.colsurfb.2010.08.048 20870397

[b24] Martinez-Gutierrez F. *et al.* Antibacterial activity, inflammatory response, coagulation and cytotoxicity effects of silver nanoparticles. Nanomedicine 8, 328–336 (2012). 21718674 10.1016/j.nano.2011.06.014

[b25] Meng J. *et al.* Using gold nanorods core/silver shell nanostructures as model material to probe biodistribution and toxic effects of silver nanoparticles in mice. Nanotoxicology 8, 686–696 (2014). 23837638 10.3109/17435390.2013.822593

[b26] Bandyopadhyay D., Baruah H., Gupta B. & Sharma S. Silver nano particles prevent platelet adhesion on immobilized fibrinogen. Indian J. Clin. Biochem. 27, 164–170 (2012). 23543820 10.1007/s12291-011-0169-4 PMC3358372

[b27] Deb S. *et al.* Surface tunability of nanoparticles in modulating platelet functions. Blood Cells Mol. Dis. 48, 36–44 (2012). 22033068 10.1016/j.bcmd.2011.09.011

[b28] Yu K. *et al.* Modulation of complement activation and amplification on nanoparticle surfaces by glycopolymer conformation and chemistry. ACS Nano 8, 7687–7703 (2014). 25106451 10.1021/nn504186b

[b29] De Paoli S. H. *et al.* The effect of protein corona composition on the interaction of carbon nanotubes with human blood platelets. Biomaterials 35, 6182–6194 (2014). 24831972 10.1016/j.biomaterials.2014.04.067

[b30] Wolfram J. *et al.* The nano-plasma interface: Implications of the protein corona. Colloid Surface B 124, 17–24 (2014). 10.1016/j.colsurfb.2014.02.035 PMC4152388 24656615

[b31] Dobrovolskaia M. A. & Neun B. W. Analysis of platelet aggregation. *NCL Method ITA-2* Version 1.1 (2009) http://ncl.cancer.gov/NCL_Method_ITA-2.1.pdf.

[b32] Xu Y. *et al.* Complement activation in factor D-deficient mice. Proc. Natl. Acad. Sci. USA 98, 14577–14582 (2001). 11724962 10.1073/pnas.261428398 PMC64724

[b33] Janssen B. J. *et al.* Structures of complement component C3 provide insights into the function and evolution of immunity. Nature 437, 505–511 (2005). 16177781 10.1038/nature04005

[b34] Liu W. *et al.* Protein corona formation for nanomaterials and proteins of a similar size: hard or soft corona? Nanoscale 5, 1658–1668 (2013). 23334428 10.1039/c2nr33611a

[b35] Winzen S. *et al.* Complementary analysis of the hard and soft protein corona: sample preparation critically effects corona composition. Nanoscale 7, 2992–3001 (2015). 25599336 10.1039/c4nr05982d

[b36] Mohanty S. *et al.* An investigation on the antibacterial, cytotoxic, and antibiofilm efficacy of starch-stabilized silver nanoparticles. Nanomedicine 8, 916–924 (2012). 22115597 10.1016/j.nano.2011.11.007

[b37] Kwon T. *et al.* Optimizing hemocompatibility of surfactant-coated silver nanoparticles in human erythrocytes. J. Nanosci. Nanotechno. 12, 6168–6175 (2012). 10.1166/jnn.2012.6433 22962723

[b38] Sopjani M., Foller M., Haendeler J., Gotz F. & Lang F. Silver ion-induced suicidal erythrocyte death. J. Appl. Toxicol. 29, 531–536 (2009). 19444854 10.1002/jat.1438

[b39] Li S. Q. *et al.* Nanotoxicity of TiO_2_ nanoparticles to erythrocyte *in vitro*. Food Chem. Toxicol. 46, 3626–3631 (2008). 18840495 10.1016/j.fct.2008.09.012

[b40] Ivask A. *et al.* Toxicity mechanisms in *Escherichia coli* vary for silver nanoparticles and differ from ionic silver. ACS Nano 8, 374–386 (2014). 24341736 10.1021/nn4044047

[b41] Almofti M. R., Ichikawa T., Yamashita K., Terada H. & Shinohara Y. Silver ion induces a cyclosporine a-insensitive permeability transition in rat liver mitochondria and release of apoptogenic cytochrome C. J. Biochem. 134, 43–49 (2003). 12944369 10.1093/jb/mvg111

[b42] Wataha J. C., Lockwood P. E., Schedle A., Noda M. & Bouillaguet S. Ag, Cu, Hg and Ni ions alter the metabolism of human monocytes during extended low-dose exposures. J. Oral Rehabil. 29, 133–139 (2002). 11856391 10.1046/j.1365-2842.2002.00845.x

[b43] AshaRani P. V., Low Kah Mun G., Hande M. P. & Valiyaveettil, S. Cytotoxicity and genotoxicity of silver nanoparticles in human cells. ACS Nano 3, 279–290 (2009). 19236062 10.1021/nn800596w

[b44] Greulich C. *et al.* Cell type-specific responses of peripheral blood mononuclear cells to silver nanoparticles. Acta Biomater. 7, 3505–3514 (2011). 21651999 10.1016/j.actbio.2011.05.030

[b45] Park E. J., Yi J., Kim Y., Choi K. & Park K. Silver nanoparticles induce cytotoxicity by a Trojan-horse type mechanism. Toxicol. In Vitro 24, 872–878 (2010). 19969064 10.1016/j.tiv.2009.12.001

[b46] Kim J. S. *et al.* Genotoxicity, acute oral and dermal toxicity, eye and dermal irritation and corrosion and skin sensitisation evaluation of silver nanoparticles. Nanotoxicology 7, 953–960 (2013). 22417112 10.3109/17435390.2012.676099

[b47] Hyun-Jeong E., Nivedita C., Jeongsoo L. & Jinhee C. Integrated mRNA and micro RNA profiling reveals epigenetic mechanism of differential sensitivity of Jurkat T cells to AgNPs and Ag ions. Toxicol. Lett. 229, 311–318 (2014). 24974767 10.1016/j.toxlet.2014.05.019

[b48] Parnsamut C. & Brimson S. Effects of silver nanoparticles and gold nanoparticles on IL-2, IL-6, and TNF-alpha production via MAPK pathway in leukemic cell lines. Genet. Mol. Res. 14, 3650–3668 (2015). 25966134 10.4238/2015.April.17.15

[b49] van der Zande M. *et al.* Distribution, elimination, and toxicity of silver nanoparticles and silver ions in rats after 28-day oral exposure. ACS Nano 6, 7427–7442 (2012). 22857815 10.1021/nn302649p

[b50] Gengan R. M., Anand K., Phulukdaree A. & Chuturgoon A. A549 lung cell line activity of biosynthesized silver nanoparticles using *Albizia adianthifolia* leaf. Colloid Surface B 105, 87–91 (2013). 10.1016/j.colsurfb.2012.12.044 23352951

[b51] Ragaseema V. M., Unnikrishnan S., Kalliyana Krishnan V. & Krishnan L. K. The antithrombotic and antimicrobial properties of PEG-protected silver nanoparticle coated surfaces. Biomaterials 33, 3083–3092 (2012). 22284585 10.1016/j.biomaterials.2012.01.005

[b52] Krishnaraj R. N. & Berchmans S. *In vitro* antiplatelet activity of silver nanoparticles synthesized using the microorganism *Gluconobacter roseus*: an AFM-based study. RSC Adv. 3, 8953–8959 (2013).

[b53] Park K. Toxicokinetic differences and toxicities of silver nanoparticles and silver ions in rats after single oral administration. J. Toxicol. Environ. Health A 76, 1246–1260 (2013). 24283396 10.1080/15287394.2013.849635

[b54] Nel A. E. *et al.* Understanding biophysicochemical interactions at the nano-bio interface. Nat. Mater. 8, 543–557 (2009). 19525947 10.1038/nmat2442

[b55] Treuel L., Docter D., Maskos M. & Stauber R. H. Protein corona - from molecular adsorption to physiological complexity. Beilstein J. Nanotechnol. 6, 857–873 (2015). 25977856 10.3762/bjnano.6.88 PMC4419682

[b56] Monopoli M. P. *et al.* Physical-chemical aspects of protein corona: relevance to *in vitro* and *in vivo* biological impacts of nanoparticles. J. Am. Chem. Soc. 133, 2525–2534 (2011). 21288025 10.1021/ja107583h

[b57] Monopoli M. P., Aberg C., Salvati A. & Dawson K. A. Biomolecular coronas provide the biological identity of nanosized materials. Nat. Nanotechnol. 7, 779–786 (2012). 23212421 10.1038/nnano.2012.207

[b58] Tenzer S. *et al.* Rapid formation of plasma protein corona critically affects nanoparticle pathophysiology. Nat. Nanotechnol. 8, 772–781 (2013). 24056901 10.1038/nnano.2013.181

[b59] Walkey C. D. *et al.* Protein corona fingerprinting predicts the cellular interaction of gold and silver nanoparticles. ACS Nano 8, 2439–2455 (2014). 24517450 10.1021/nn406018q

[b60] Jilma-Stohlawetz P. *et al.* Fy phenotype and gender determine plasma levels of monocyte chemotactic protein. Transfusion 41, 378–381 (2001). 11274594 10.1046/j.1537-2995.2001.41030378.x

[b61] Kawamoto R. *et al.* Association between fasting plasma glucose and high-sensitivity C-reactive protein: gender differences in a Japanese community-dwelling population. Cardiovasc. Diabetol. 10, 51, 10.1186/1475-2840-10-51 (2011). 21663637 PMC3135517

[b62] Nanjappa V. *et al.* Plasma Proteome Database as a resource for proteomics research: 2014 update. Nucleic Acids Res. 42, D959–965 (2014). 24304897 10.1093/nar/gkt1251 PMC3965042

[b63] Aggarwal P., Hall J. B., McLeland C. B., Dobrovolskaia M. A. & McNeil S. E. Nanoparticle interaction with plasma proteins as it relates to particle biodistribution, biocompatibility and therapeutic efficacy. Adv. Drug Deliver. Rev. 61, 428–437 (2009). 10.1016/j.addr.2009.03.009 PMC3683962 19376175

[b64] Patel H. M. Serum opsonins and liposomes - their interaction and opsonophagocytosis. Crit. Rev. Ther. Drug Carrier Syst. 9, 39–90 (1992). 1544174

[b65] Brandt J. T. Current concepts of coagulation. Clin. Obstet. Gynecol. 28, 3–14 (1985). 3886248 10.1097/00003081-198528010-00002

[b66] Truedsson L. Classical pathway deficiencies - A short analytical review. Mol. Immunol. 68, 14–19 (2015). 26038300 10.1016/j.molimm.2015.05.007

[b67] Revak S. D., Cochrane C. G. & Griffin J. H. The binding and cleavage characteristics of human Hageman factor during contact activation. A comparison of normal plasma with plasmas deficient in factor XI, prekallikrein, or high molecular weight kininogen. J. Clin. Invest. 59, 1167–1175 (1977). 864009 10.1172/JCI108741 PMC372330

[b68] Gob E. *et al.* Blocking of plasma kallikrein ameliorates stroke by reducing thromboinflammation. Ann. Neurol. 77, 784–803 (2015). 25628066 10.1002/ana.24380

[b69] Chavakis T. *et al.* Inhibition of platelet adhesion and aggregation by a defined region (Gly-486-Lys-502) of high molecular weight kininogen. J. Biol. Chem. 277, 23157–23164 (2002). 11970955 10.1074/jbc.M202529200

[b70] Chavakis T., Pixley R. A., Isordia-Salas I., Colman R. W. & Preissner K. T. A novel antithrombotic role for high molecular weight kininogen as inhibitor of plasminogen activator inhibitor-1 function. J. Biol. Chem. 277, 32677–32682 (2002). 12082110 10.1074/jbc.M204010200

[b71] Chavakis T. & Preissner K. T. Potential pharmacological applications of the antithrombotic molecule high molecular weight kininogen. Curr. Vasc. Pharmacol. 1, 59–64 (2003). 15320853 10.2174/1570161033386790

[b72] Vuilleumier N., Dayer J. M., von Eckardstein A. & Roux-Lombard P. Pro- or anti-inflammatory role of apolipoprotein A-1 in high-density lipoproteins? Swiss Med. Wkly. 143, w13781; 10.4414/smw.2013.13781 (2013). 23740387

[b73] Riddell D. R. & Owen J. S. Inhibition of ADP-induced platelet aggregation by apoE is not mediated by membrane cholesterol depletion. Thromb. Res. 81, 597–606 (1996). 9054055 10.1016/0049-3848(96)87301-4

[b74] Riddell D. R., Graham A. & Owen J. S. Apolipoprotein E inhibits platelet aggregation through the L-arginine:nitric oxide pathway. Implications for vascular disease. J. Biol. Chem. 272, 89–95 (1997). 8995232 10.1074/jbc.272.1.89

[b75] Dobrovolskaia M. A. & Neun B. W. Analysis of hemolytic properties of nanoparticles. *NCL Method ITA-1* Version 1.1, (2009). http://ncl.cancer.gov/NCL_Method_ITA-1.pdf.

[b76] Dobrovolskaia M. A. & Neun B. W. Qualitative analysis of total complement activation by Western blot. *NCL Method ITA-5.1* Version 1.1, (2010). http://ncl.cancer.gov/NCL_Method_ITA-5.1.pdf. 10.1007/978-1-60327-198-1_25 21116973

[b77] Lundqvist M. *et al.* Nanoparticle size and surface properties determine the protein corona with possible implications for biological impacts. Proc. Natl. Acad. Sci. USA 105, 14265–14270 (2008). 18809927 10.1073/pnas.0805135105 PMC2567179

[b78] McDonald W. H., Ohi R., Miyamoto D. T., Mitchison T. J. & Yates Iii J. R. Comparison of three directly coupled HPLC MS/MS strategies for identification of proteins from complex mixtures: single-dimension LC-MS/MS, 2-phase MudPIT, and 3-phase MudPIT. Int. J. Mass spectrom. 219, 245–251 (2002).

[b79] Rappsilber J., Mann M. & Ishihama Y. Protocol for micro-purification, enrichment, pre-fractionation and storage of peptides for proteomics using StageTips. Nat. Protoc. 2, 1896–1906 (2007). 17703201 10.1038/nprot.2007.261

[b80] Cox J. *et al.* Accurate proteome-wide label-free quantification by delayed normalization and maximal peptide ratio extraction, termed MaxLFQ. Mol. Cell Proteomics 13, 2513–2526 (2014). 24942700 10.1074/mcp.M113.031591 PMC4159666

[b81] Cox J. *et al.* Andromeda: a peptide search engine integrated into the MaxQuant environment. J. Proteome Res. 10, 1794–1805 (2011). 21254760 10.1021/pr101065j

[b82] R Development Core Team, R: A Language and Environment for Statistical Computing. Vienna, Austria: the R Foundation for Statistical Computing. ISBN: 3-900051-07-0 (2011). http://www.R-project.org/.

[b83] Saito R. *et al.* A travel guide to Cytoscape plugins. Nat. Methods 9, 1069–1076 (2012). 23132118 10.1038/nmeth.2212 PMC3649846

[b84] Bindea G. *et al.* ClueGO: a Cytoscape plug-in to decipher functionally grouped gene ontology and pathway annotation networks. Bioinformatics 25, 1091–1093 (2009). 19237447 10.1093/bioinformatics/btp101 PMC2666812

[b85] Boyle E. I. *et al.* GO::TermFinder--open source software for accessing Gene Ontology information and finding significantly enriched Gene Ontology terms associated with a list of genes. Bioinformatics 20, 3710–3715 (2004). 15297299 10.1093/bioinformatics/bth456 PMC3037731

