# Supplementary material for: An Evaluation of Blood Compatibility of Silver Nanoparticles
Source: Sci Rep. 2016 May 5;6:25518. doi: 10.1038/srep25518 (PMC4857076; doi:10.1038/srep25518)
Supplement: Supplementary Information [file srep25518-s1.pdf]

Supplementary Information

An Evaluation of Blood Compatibility of Silver Nanoparticles

He Huang [1, †], Wenjia Lai [2, †], Menghua Cui [1], Ling Liang [1, 3], Yuchen Lin [2], Qiaojun Fang [2,*], Ying Liu [2,*], Liming Xie [1]

[1] Key Laboratory of Standardization and Measurement for Nanotechnology of Chinese Academy of Sciences, CAS Center for Excellence in Nanoscience, National Center for Nanoscience and Technology, Beijing 100190, P. R. China.

[2] Key Laboratory for Biomedical Effects of Nanomaterials and Nanosafety of Chinese Academy of Sciences, CAS Center for Excellence in Nanoscience, National Center for Nanoscience and Technology, Beijing 100190, P. R. China.

[3] College of Chemistry and Chemical Engineering, Lanzhou University, Lanzhou, 730000, P. R. China.

[†] Contributed equally to this work.

*Corresponding author: Qiaojun Fang, Tel: +86-10-82545562, Fax: +86-10-82545643, E-mail: fangqj@nanoctr.cn; Ying Liu, Tel: +86-010-82545665, E-mail: liuy1@nanoctr.cn.

Figure S1: Lymphocyte proliferation after citrate and Ag ion treatment

Figure S2: Effect of AgNPs at high concentrations on platelet aggregation and coagulation

Figure S3: The full-length gel images of qualitative analysis of total complement activation by Western blotting

Figure S4: Protein coronas and their compositions of AgNPs

Figure S5: Correlations of label-free quantification intensities depicting the reproducibility across two biological replicates of AgNPs corona proteomes

Figure S6: Relative plasma protein concentrations and protein concentrations

Figure S7: Relative pI and MW of plasma proteins and corona proteins

Table S1: List of plasma proteins on AgNPs corona

Table S2: Correlation analysis of corona proteins' pI and MW with their label-free quantification intensities.

Table S3: All enriched GO biological processes for identified corona proteins

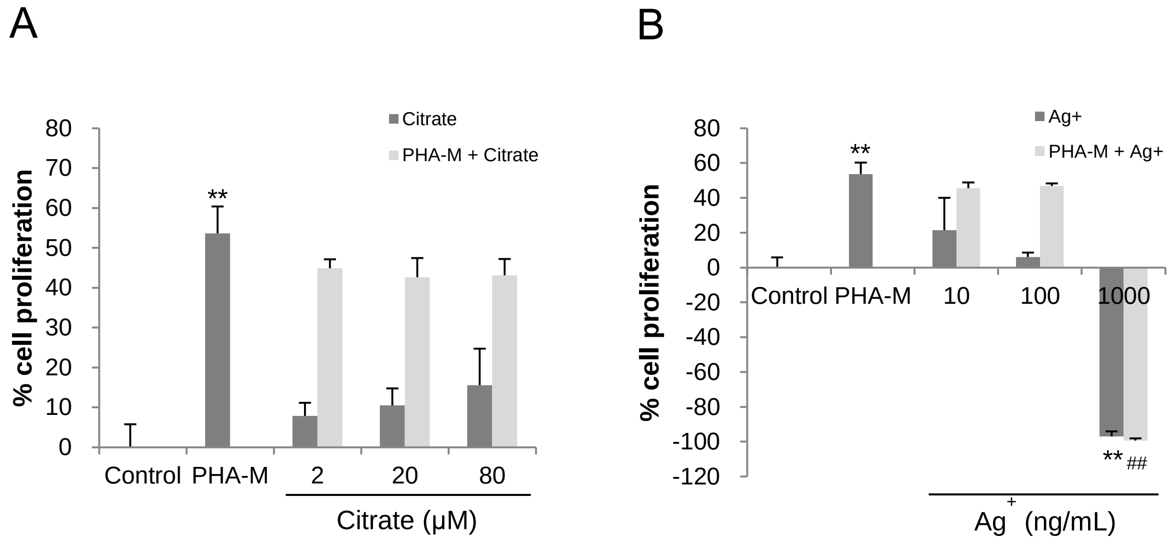

Figure S1. Lymphocyte proliferation after citrate and Ag ion treatment. PBMCs were treated with different concentrations of citrate (A) or Ag ion (B) for 3 days, and then measured by MTS assay. RPMI-1640 and PHA-M were used as negative control and positive control, respectively. ***p* < 0.01, significantly different from negative control. #*p* < 0.05, ##*p* < 0.01, significantly different from positive control.

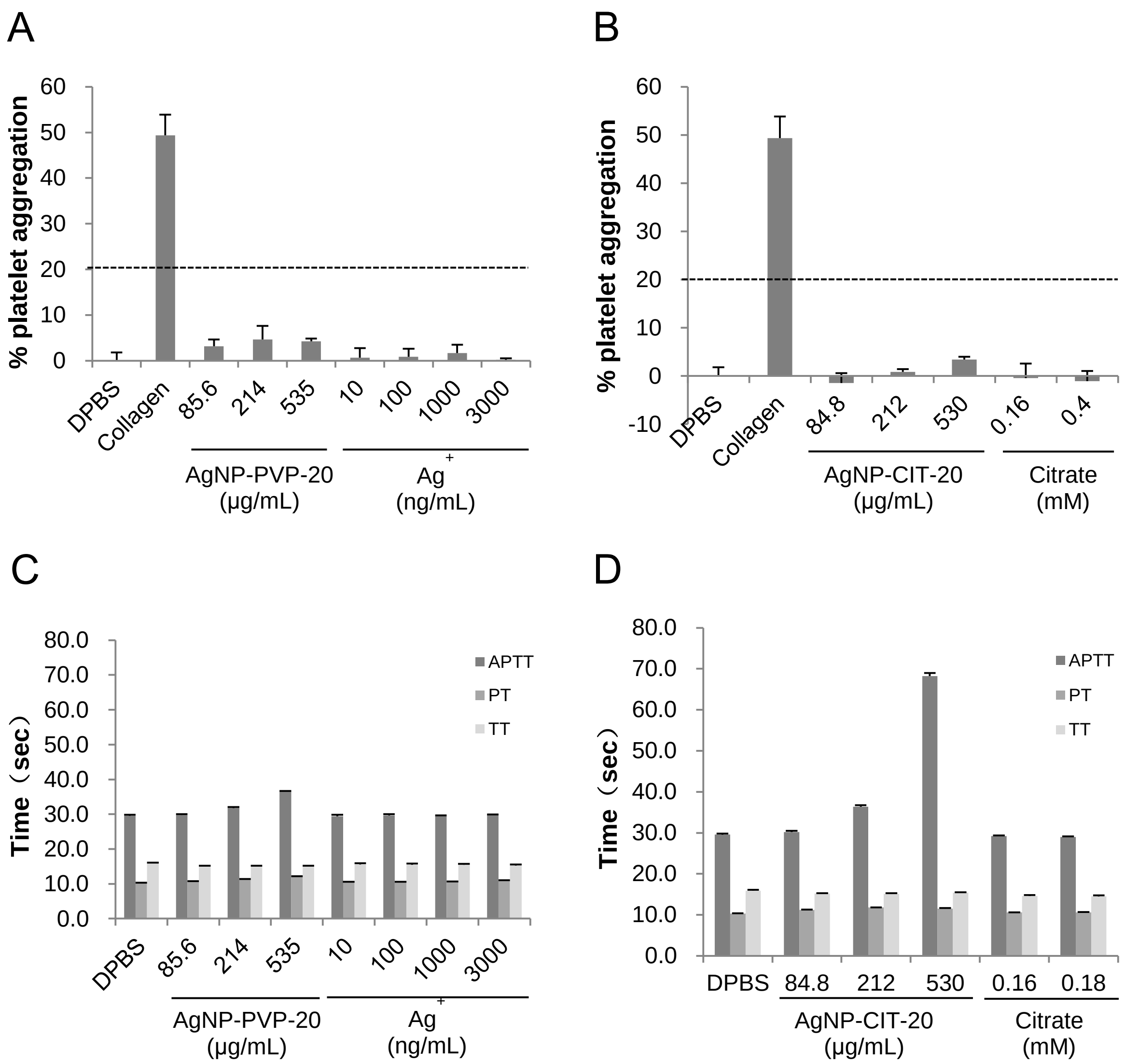

Figure S2. Effect of AgNPs at high concentrations on platelet aggregation and coagulation. Platelet aggregation was measured by incubating PRP with AgNP-PVP-20 (A) or AgNP-CIT-20 (B) for 15 min. 1 × DPBS and 1.0 mg/mL collagen were used as negative control and positive control, respectively. 20% of platelet aggregation was defined as the assay threshold (dash line). In the coagulation assay, APTT, PT and TT were separately tested after exposure PPP to AgNP-PVP-20 (C) and AgNP-CIT-20 (D) for 30 min. In addition, different concentrations of $AgNO_3$ as well as citrate were also included.

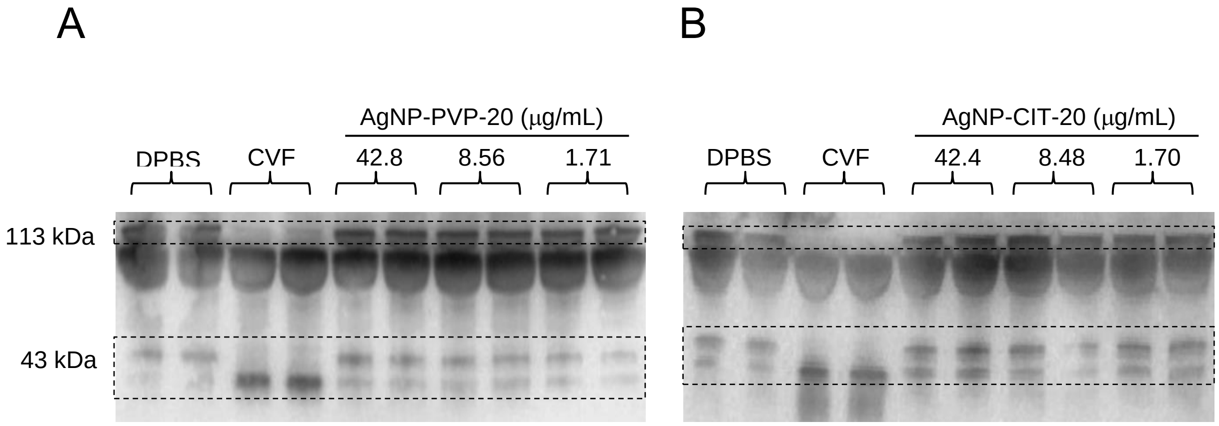

Figure S3. The full-length gel images of qualitative analysis of total complement activation by Western blotting. The expression levels of C3-α chain (115 kDa) and cleavage products (~40 kDa) were determined by Western blotting analysis after exposure to human normal plasma to indicated concentrations of AgNPs for 30 min at 37°C. 1 × DPBS and CVF were used as negative control and positive control, respectively. The representative full-length immunoblotting images of AgNP-PVP-20 (A) and AgNP-CIT-20 (B) were presented.

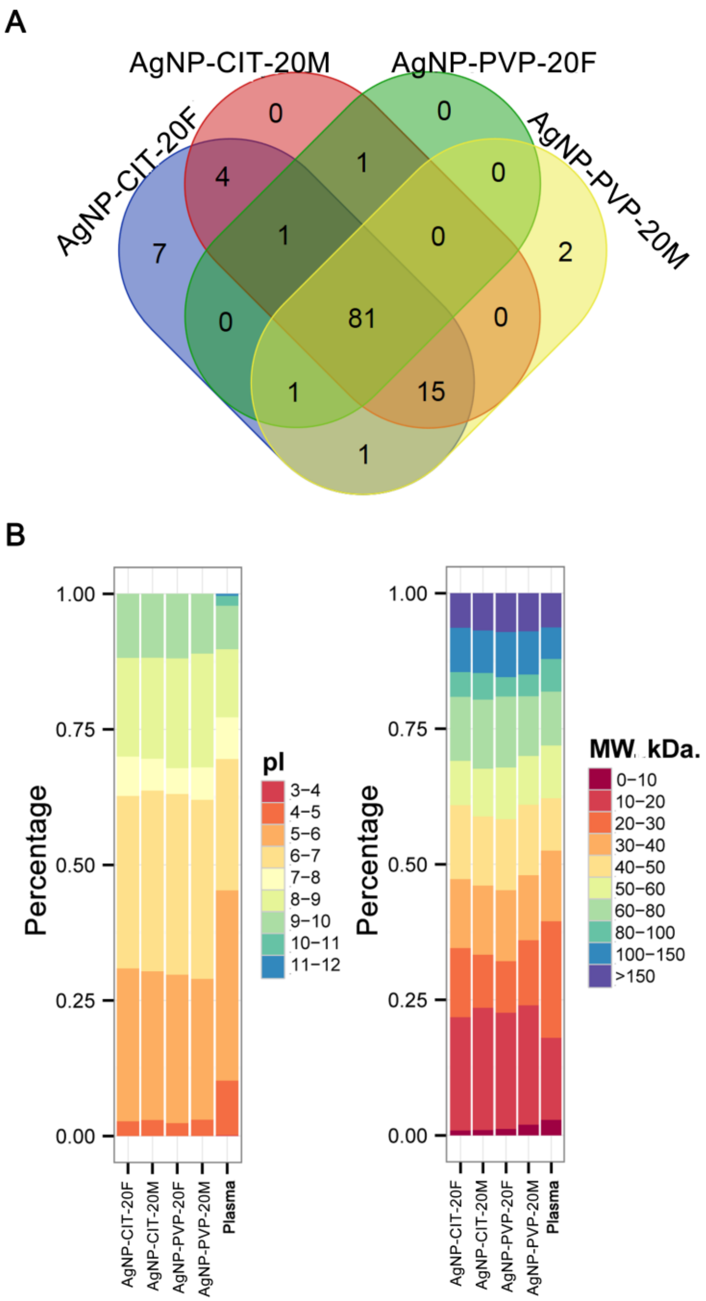

Figure S4. Protein coronas and their compositions of AgNPs. (A) Overlaps of proteins identifications from citrate or PVP coated AgNPs incubated with female or male plasma by label-free quantification. Numbers of proteins identified in both biological replicates of each approach were shown. Classification of plasma proteins and corona proteins identified on citrate or PVP coated AgNPs by LC-MS according to the MW (B) and pI (C).

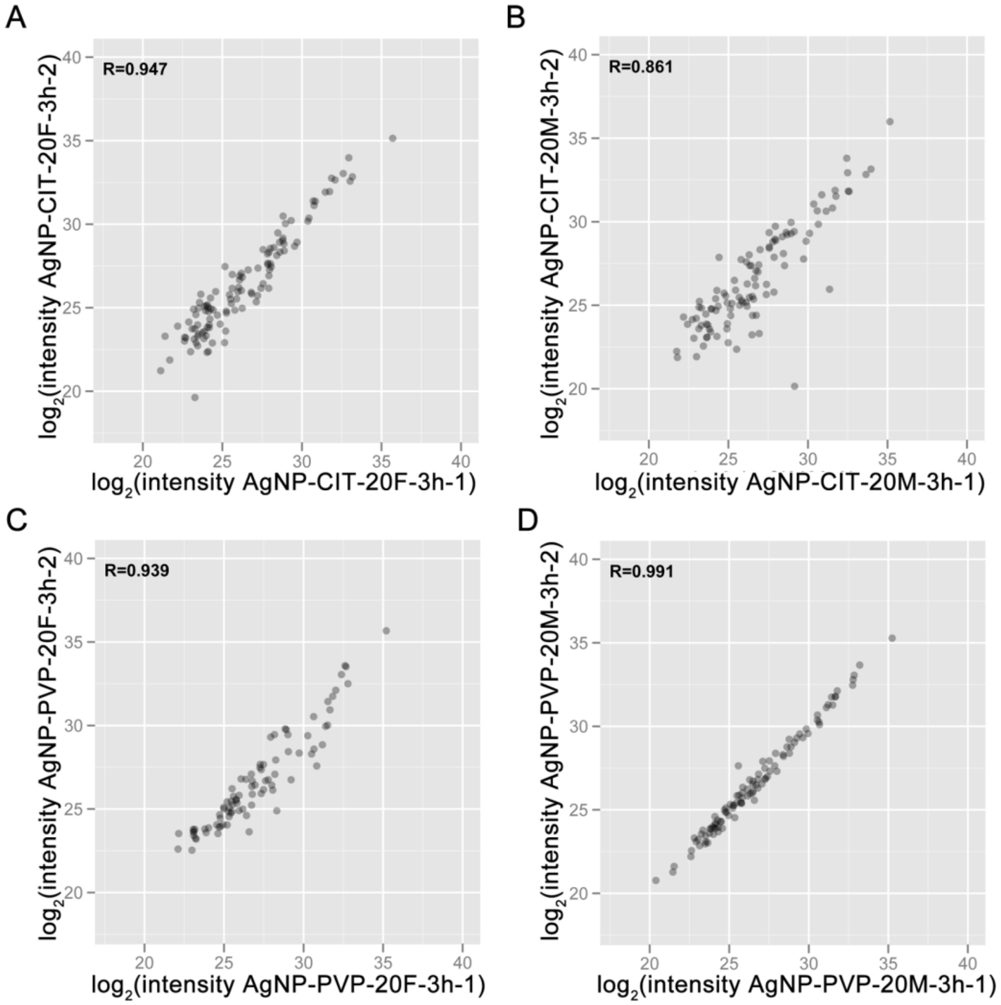

Figure S5. Correlations of label-free quantification intensities depicting the reproducibility across two biological replicates of AgNPs corona proteomes. (A) AgNP-CIT-20F, (B) AgNP-CIT-20M, (C) AgNP-PVP-20F and (D) AgNP-PVP-20M.

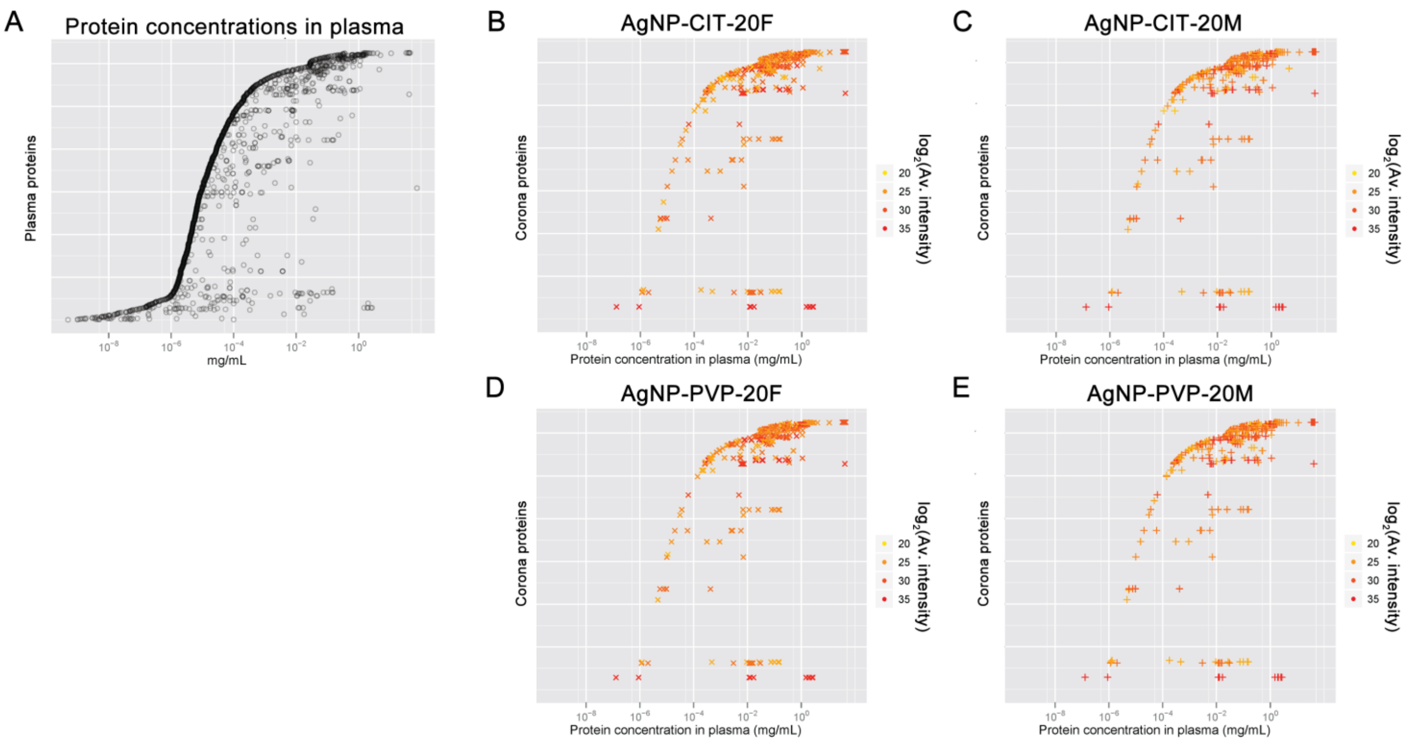

Figure S6. Relative plasma protein concentrations (A) and protein concentrations as well as intensities of corona proteins identified in four experiments by LC-MS and their label-free quantification. (B) AgNP coated with citrate using female plasma sample, (C) AgNP coated with citrate using male plasma sample (D) AgNP coated with PVP using female plasma sample and (E) AgNP coated with PVP using male plasma sample. Y axis represents different proteins and x axis is different concentrations observed for each protein. As in Plasma Proteome Database (PPD), one protein usually has several concentrations. Intensities in log 2 scales are divided in four different categories as indicated by different colors (red for 35, orange for 30, dark yellow for 30 and bright yellow for 25).

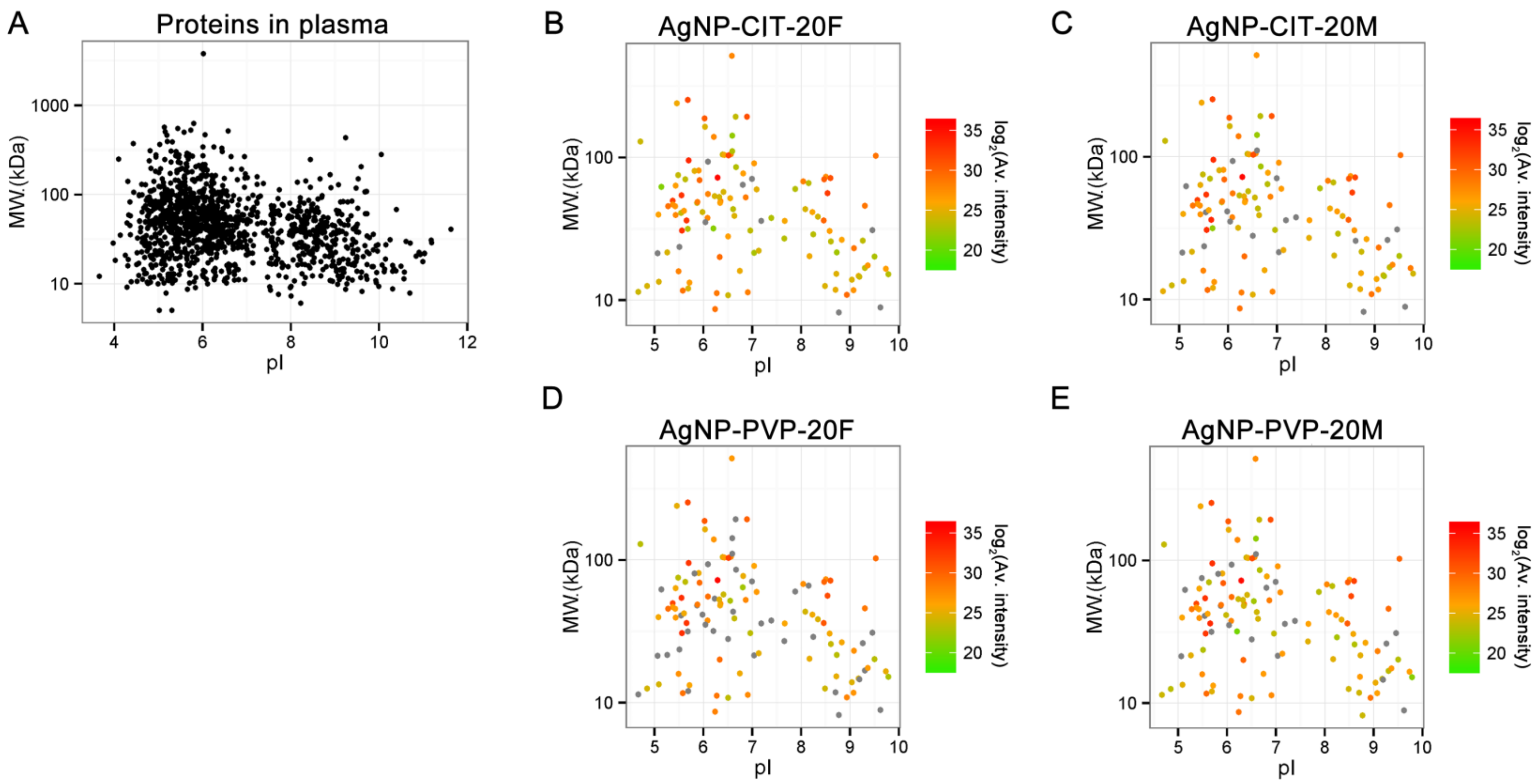

Figure S7. Relative pI and MW of plasma proteins (A) and corona proteins identified in (B) AgNP-CIT-20F, (C) AgNP-CIT-20M, (D) AgNP-PVP-20F and (E) AgNP-PVP-20M experiments with their label-free quantification intensities colored in green to red gradient showing low to high intensity accordingly.

Table S1. List of plasma proteins on AgNPs corona identified by LC-MS, including log_2 transformed average (Av.) intensity on label free quantitative proteomics

Uniprot ID	log_2 Av. (intensity AgNP-CIT-20F-3h)	log_2 Av. (intensity AgNP-CIT-20M-3h)	log_2 Av. (intensity AgNP-PVP-20F-3h)	log_2 Av. (intensity AgNP-PVP-20M-3h)	log_2 Av. (intensity blank)	Mol. weight [kDa]	Isoelectric Point (pI)	Gene names	Protein names
B7ZKJ8	25.611	25.559	26.435	25.243		103.880	6.430	ITIH4	35 kDa inter-alpha-trypsin inhibitor heavy chain H4
P00568	22.344	23.943	23.221	24.940		21.635	8.730	AK1	Adenylate kinase isoenzyme 1
P01009	27.305	26.822	26.634	26.993		46.736	5.370	SERPINA1	Alpha-1-antitrypsin
P08697-2	23.625	24.029				47.906	5.870	SERPINF2	Alpha-2-antiplasmin
P02765	27.720	26.904	26.547	27.083		39.324	5.430	AHSG	Alpha-2-HS-glycoprotein
P01023	24.615	24.246	25.092	25.195		163.290	6.030	A2M	Alpha-2-macroglobulin
P37840-2	23.987	25.082		24.001		11.372	4.670	SNCA	Alpha-synuclein
P03950	26.372	28.429	25.106	26.299		16.550	9.730	ANG	Angiogenin
P01008	24.266	25.121	24.935	24.327		52.602	6.320	SERPINC1	Antithrombin-III
P02647	33.006	33.120	33.097	32.608	30.833	30.777	5.560	APOA1	Apolipoprotein A-I
P02652	28.646	28.439	28.813	28.307		11.175	6.270	APOA2	Apolipoprotein A-II
P06727	29.110	28.839	29.318	28.585		45.398	5.280	APOA4	Apolipoprotein A-IV
Q6Q788	24.074			23.843		41.212	5.980	APOA5	Apolipoprotein A-V
P04114	27.755	26.932	26.692	27.443		515.600	6.580	APOB	Apolipoprotein B-100
P02654	28.983	29.114	28.092	29.067		9.332	8.010	APOC1	Apolipoprotein C-I
P02655	29.305	29.449	29.335	29.759		11.284	4.640	APOC2	Apolipoprotein C-II
P55056	24.020	23.150				14.553	9.190	APOC4	Apolipoprotein C-IV
P02649	32.803	33.554	32.716	33.434		36.154	5.650	APOE	Apolipoprotein E
O14791-3	26.472	25.934	25.512	26.001		42.158	5.600	APOL1	Apolipoprotein L1
Q9BXN1	23.371	23.643		24.134		43.417	6.610	ASPN	Asporin
P02749	24.236	24.958	24.604	25.161		38.298	8.340	APOH	Beta-2-glycoprotein 1
H3BVI7	27.038	27.162	26.330	26.138		45.520	5.420	CDH1	Cadherin-1
P15169	28.474	27.989	27.637	28.339		52.286	6.860	CPN1	Carboxypeptidase N catalytic chain
P10909-4	28.421	28.275	28.617	28.824		48.803	5.880	CLU	Clusterin
P12259	31.697	31.791	31.482	31.737		251.700	5.680	F5	Coagulation factor V
P03951	30.276	30.712	29.826	30.388		70.108	8.470	F11	Coagulation factor XI
P00748	28.276	28.295	27.266	27.725		67.791	8.040	F12	Coagulation factor XII
Q9Y6Z7	23.517	23.346	23.384	23.563		30.705	6.960	COLEC10	Collectin-10
Q9BWP8-8	24.475	24.649		24.953		21.589	5.260	COLEC11	Collectin-11
P02745	23.493					26.016	9.260	C1QA	Complement C1q subcomponent subunit A
P02746	25.966	25.895	25.676	25.741		26.722	8.830	C1QB	Complement C1q subcomponent subunit B
P02747	23.464		23.797	22.997		25.773	8.610	C1QC	Complement C1q subcomponent subunit C

Uniprot ID	log2 Av. (intensity AgNP-CIT-20F-3h)	log2 Av. (intensity AgNP-CIT-20M-3h)	log2 Av. (intensity AgNP-PVP-20F-3h)	log2 Av. (intensity AgNP-PVP-20M-3h)	log2 Av. (intensity blank)	Mol. weight [kDa]	Isoelectric Point (pI)	Gene names	Protein names
P00736	24.971	24.795				80.118	5.820	C1R	Complement C1r subcomponent
P01024	30.401	30.619	30.581	30.613		187.150	6.020	C3	Complement C3
P0C0L4	23.650	23.375		23.850		192.780	6.660	C4A	Complement C4-A
P0C0L5	31.106	30.887	30.015	31.115		192.750	6.890	C4B	Complement C4-B
P13671	23.962	23.872	24.622	24.025		104.790	6.390	C6	Complement component C6
P07358	23.124	22.995		23.224		67.047	8.500	C8B	Complement component C8 beta chain
P02748	26.335	25.168	25.588	25.762		63.173	5.430	C9	Complement component C9
P00751	23.042	22.460		23.009		85.532	6.670	CFB	Complement factor B
P00746	23.170	25.032		24.839		27.033	7.650	CFD	Complement factor D
P08603	27.620	28.453	26.821	27.655		139.090	6.210	CFH	Complement factor H
Q03591	22.948					37.650	7.390	CFHR1	Complement factor H-related protein 1
Q9BXR6		23.238	22.355			64.419	6.800	CFHR5	Complement factor H-related protein 5
P02671	33.461	33.238	32.645	32.956	31.096	94.972	5.700	FGA	Fibrinogen alpha chain
P02675	32.366	32.180	31.297	31.592	28.998	55.928	8.540	FGB	Fibrinogen beta chain
P02679-2	32.309	32.199	31.786	31.710	29.121	49.496	5.370	FGG	Fibrinogen gamma chain
P02751-10	25.510	25.578	25.042	25.585		239.620	5.460	FN1	Fibronectin
O75636-2	21.451	24.653		20.581		31.677	6.200	FCN3	Ficolin-3
P30043	24.383	25.455	25.001	26.546		22.119	7.130	BLVRB	Flavin reductase (NADPH)
P06396-2	26.941	26.630	25.308	25.446		80.640	5.900	GSN	Gelsolin
P00738	23.710					45.205	6.130	HP	Haptoglobin
P00739	24.622	24.450	23.730	24.390		39.029	6.630	HPR	Haptoglobin-related protein
P69905	24.725	25.574	25.068	26.503		15.257	8.720	HBA1	Hemoglobin subunit alpha
P68871	25.271	25.557	25.401	26.544		15.998	6.740	HBB	Hemoglobin subunit beta
P02790	25.606	24.070	22.841	22.397		51.676	6.550	HPX	Hemopexin
P05546	24.449	23.707	23.954	24.389		57.070	6.410	SERPIND1	Heparin cofactor 2
P04196	24.919	26.513	26.606	27.198		59.578	7.090	HRG	Histidine-rich glycoprotein
P01876	27.828	28.028	27.347	27.746		37.654	6.080	IGHA1	Ig alpha-1 chain C region
P01857	29.768	30.252	30.669	30.446		36.105	8.460	IGHG1	Ig gamma-1 chain C region
P01859	25.711	26.230	25.861	26.075		35.900	7.660	IGHG2	Ig gamma-2 chain C region
P01860	25.887	26.440	25.423	26.604		41.287	8.230	IGHG3	Ig gamma-3 chain C region
P01781	25.025	25.196		24.148		11.771	8.700		Ig heavy chain V-III region GAL
P01764	24.606	24.416	24.074	24.153		12.582	8.490		Ig heavy chain V-III region VH26
P01834	28.988	29.360	29.036	29.472		11.609	5.580	IGKC	Ig kappa chain C region
P01617	24.646	25.227		24.815		12.059	5.690		Ig kappa chain V-II region TEW
P04433	24.403	24.020	24.299	23.314		12.575	4.850		Ig kappa chain V-III region VG
P01623	26.998	26.765	26.901	26.235		11.746	9.070		Ig kappa chain V-III region WOL
P06312	24.552	24.764	24.368	24.820		13.380	5.090	IGKV4-1	Ig kappa chain V-IV region
P0CG05	28.285	27.873	27.574	27.506		11.293	6.910	IGLC2	Ig lambda-2 chain C regions

Uniprot ID	log$_2$ Av. (intensity AgNP-CIT-20F-3h)	log$_2$ Av. (intensity AgNP-CIT-20M-3h)	log$_2$ Av. (intensity AgNP-PVP-20F-3h)	log$_2$ Av. (intensity AgNP-PVP-20M-3h)	log$_2$ Av. (intensity blank)	Mol. weight [kDa]	Isoelectric Point (pI)	Gene names	Protein names
P01871	28.126	28.742	27.097	28.698		49.306	6.350	IGHM	Ig mu chain C region
C9JA05				23.970		8.168	8.770	IGJ	Immunoglobulin J chain
B9A064	28.847	28.957	27.215	28.148		23.063	9.080	IGLL5	Immunoglobulin lambda-like polypeptide 5
P05019-3	23.183	24.204	22.760	21.367		15.177	9.780	IGF-I	Insulin-like growth factor I
P01344	22.812	22.929	23.256	23.142		20.140	9.500	IGF2	Insulin-like growth factor II
P18065	25.401	24.354	25.170	24.849		20.281	8.170	IGFBP2	Insulin-like growth factor-binding protein 2
P17936	26.796	27.659	26.575	26.800		31.674	9.030	IGFBP3	Insulin-like growth factor-binding protein 3
P24593	26.366	26.862	25.585	25.579		30.570	8.580	IGFBP5	Insulin-like growth factor-binding protein 5
Q16270-2	23.250	23.439		22.593		28.860	8.250	IGFBP7	Insulin-like growth factor-binding protein 7
P19823	27.760	27.162	27.469	27.038		106.463	6.400	ITIH2	Inter-alpha-trypsin inhibitor heavy chain H2
Q14624	31.846	31.184	32.066	31.254		103.360	6.510	ITIH4	Inter-alpha-trypsin inhibitor heavy chain H4
P01042-2	26.241	26.139	25.650	24.231		47.883	6.340	KNG1	Isoform LMW of Kininogen-1
P35527	21.177					62.064	5.140	KRT9	Keratin, type I cytoskeletal 9
P04264	23.419	23.598		23.402		66.038	8.150	KRT1	Keratin, type II cytoskeletal 1
P01042	35.422	35.570	35.432	35.258		71.957	6.290	KNG1	Kininogen-1
P02788-2	27.422	26.857	27.371	26.590		73.160	8.500	LTF	Lactotransferrin
P18428	24.440	23.693		23.978		53.383	6.230	LBP	Lipopolysaccharide-binding protein
Q13201	22.928					138.110	8.150	MMRN1	Multimerin-1
P03952	31.054	31.642	30.768	31.957		71.369	8.600	KLKB1	Plasma kallikrein
P05155	28.019	28.657	29.203	29.265		55.154	6.090	SERPING1	Plasma protease C1 inhibitor
P05154	28.755	28.747	28.739	29.489		45.674	9.300	SERPINA5	Plasma serine protease inhibitor
P00747	26.628	26.898	25.982	26.811		90.568	7.040	PLG	Plasminogen
P02775	24.557	25.677	24.691	25.291		13.894	9.040	PPBP	Platelet basic protein
P02776	29.501	28.997	27.983	28.995		10.845	8.930	PF4	Platelet factor 4
P02760	22.684					38.999	5.950	AMBP	Protein AMBP
P05109	24.048	24.858	23.474	24.348		10.834	6.500	S100A8	Protein S100-A8
P06702	26.491	26.822	25.883	26.187		13.242	5.710	S100A9	Protein S100-A9
Q92954-5	28.904	29.271	29.233	29.842		102.510	9.530	PRG4	Proteoglycan 4
P00734	23.498	23.368	23.425	23.341		70.036	5.630	F2	Prothrombin
P34096	23.629	23.545		23.477		16.840	9.300	RNASE4	Ribonuclease 4
P49908	24.440	25.532	24.298	26.605		43.353	8.080	SEPP1	Selenoprotein P
P02787	26.306	25.972	25.201	25.669		77.063	6.810	TF	Serotransferrin
P02768	29.650	29.701	29.399	30.457	27.803	69.366	5.920	ALB	Serum albumin
P35542	25.320	24.366	24.501	23.770		14.746	9.170	SAA4	Serum amyloid A-4 protein
P27169	26.110	25.643	25.659	25.923		39.731	5.080	PON1	Serum paraoxonase/arylesterase 1
P04278-5	23.082	21.835				41.712	6.220	SHBG	Sex hormone-binding globulin
Q8TER0-5	21.780	21.997		21.576		142.070	6.590	SNED1	Sushi, nidogen and EGF-like domain-containing protein 1
P07996	23.941	23.748	23.226	23.680		129.380	4.710	THBS1	Thrombospondin-1

Uniprot ID	log_2 Av. (intensity AgNP-CIT-20F-3h)	log_2 Av. (intensity AgNP-CIT-20M-3h)	log_2 Av. (intensity AgNP-PVP-20F-3h)	log_2 Av. (intensity AgNP-PVP-20M-3h)	log_2 Av. (intensity blank)	Mol. weight [kDa]	Isoelectric Point (pI)	Gene names	Protein names
Q15582				23.060		74.681	7.620	TGFBI	Transforming growth factor-beta-induced protein ig-h3
P02766	28.030	27.951	27.150	27.363		15.887	5.490	TTR	Transthyretin
P07225	23.728	23.764	23.350			75.122	5.480	PROS1	Vitamin K-dependent protein S
P04004	32.813	32.714	33.092	32.789		54.305	5.550	VTN	Vitronectin
Q6ZN30	26.312					122.330	6.070	BNC2	Zinc finger protein basonuclin-1

Table S2. Correlation analysis of corona proteins' pI and MW with their label-free quantification intensities.

		AgNP-CIT-20F	AgNP-CIT-20M	AgNP-PVP-20F	AgNP-PVP-20M
pI	Correlation Coefficient	-0.138	-0.105	-0.158	-0.146
	p value*	0.151	0.295	0.152	0.146
MW	Correlation Coefficient	0.119	0.078	0.204	0.163
	p value*	0.215	0.433	0.063	0.106

*Two tailed Spearman test was used.

Table S3. All enriched GO biological processes for identified corona proteins

GO ID	GO Term	GO Levels	Nr. Genes	% Associated Genes	Term p -Value Corrected with Bonferroni step down	Associated Genes Found
GO:0002253	activation of immune response	[2, 4, 5, 6]	30	5.1457977	5.59E-17	[A2M, C1QA, C1QB, C1QC, C1R, C3, C4A, C4B, C6, C8B, C9, CFB, CFD, CFH, CFHR1, CFHR5, CLU, FCN3, IGHG1, IGHG2, IGHG3, IGKC, IGKV4-1, IGLC2, KRT1, LBP, LTF, PROS1, SERPING1, VTN]
GO:0002526	acute inflammatory response	[5]	26	17.931034	1.90E-28	[A2M, AHSG, APOA2, C3, C4A, C4B, C6, C8B, C9, CFB, CFH, F12, F2, FN1, HP, HPR, ITIH4, KLKB1, LBP, PROS1, S100A8, SAA4, SERPINA1, SERPINF2, SERPING1, VTN]
GO:0006953	acute-phase response	[6]	10	18.181818	8.71E-10	[AHSG, F2, FN1, HP, HPR, ITIH4, LBP, SAA4, SERPINA1, SERPINF2]
GO:0002250	adaptive immune response	[3]	21	5.357143	1.85E-11	[C1QA, C1QB, C1QC, C1R, C3, C4A, C4B, C6, C8B, C9, CLU, HPX, IGHG1, IGHG2, IGHG3, IGHM, IGJ, IGKC, IGKV4-1, IGLC2, SERPING1]
GO:0043277	apoptotic cell clearance	[4, 5, 6, 7]	5	13.888889	0.001733726	[C3, C4A, C4B, FCN3, THBS1]
GO:0007596	blood coagulation	[4, 5]	38	6.5404477	2.62E-26	[A2M, ALB, APOA1, APOB, APOE, APOH, CFD, CLU, F11, F12, F2, F5, FGA, FGB, FGG, FN1, HBB,HRG, IGF1, IGF2, KLKB1, KNG1, KRT1, MMRN1, PF4, PLG, PPBP, PROS1, SEPP1, SERPINA1,SERPINA5, SERPINC1, SERPIND1, SERPINF2, SERPING1, TF, THBS1, VTN]
GO:0072378	blood coagulation, fibrin clot formation	[3, 4, 5, 6]	10	37.037037	2.97E-13	[A2M, APOH, F11, F12, F2, FGA, KLKB1, KNG1, SERPINC1, SERPING1]
GO:0001775	cell activation	[3]	33	3.4303534	1.10E-13	[A2M, ALB, APOA1, APOE, CFD, CLU, F2, F5, FGA, FGB, FGG, FN1, HBB, HRG, IGF1, IGF2, IGFBP2,KNG1, LBP, MMRN1, PF4, PLG, PPBP, PROS1, S100A8, S100A9, SEPP1, SERPINA1, SERPINF2, SERPING1, SNCA, TF, THBS1]
GO:0016477	cell migration	[3, 4, 5]	22	1.9147085	0.001025016	[ANG, APOA1, APOB, APOE, APOH, CDH1, F2, FN1, HRG, IGF1, IGFBP3, IGFBP5, LBP, PF4, PLG, PPBP, PROS1, S100A8, S100A9, SEPP1, THBS1, VTN]
GO:0048878	chemical homeostasis	[4]	21	2.1276596	3.49E-04	[APOA1, APOA2, APOA4, APOA5, APOB, APOC2, APOC4, APOE, CDH1, CFH, F2, HPX, IGF1, IGFBP5, KNG1, KRT1, LTF, S100A8, S100A9, SNCA, TF]
GO:0042632	cholesterol homeostasis	[7]	7	9.589041	2.22E-04	[APOA1, APOA2, APOA4, APOA5, APOB, APOC2, APOE]
GO:0008203	cholesterol metabolic process	[6]	8	6.0150375	0.001103614	[APOA1, APOA2, APOA4, APOA5, APOB, APOC1, APOE, APOL1]
GO:0030301	cholesterol transport	[5, 6, 7]	10	13.513514	1.91E-08	[APOA1, APOA2, APOA4, APOA5, APOB, APOC1, APOC2, APOE, CLU, PON1]
GO:0034382	chylomicron remnant clearance	[5, 6]	3	42.857143	0.005076655	[APOC1, APOC2, APOE]
GO:0006956	complement activation	[3, 4, 5, 6, 7]	28	14.893617	1.78E-28	[A2M, C1QA, C1QB, C1QC, C1R, C3, C4A, C4B, C6, C8B, C9, CFB, CFD, CFH, CFHR1, CFHR5, CLU, FCN3, IGHG1, IGHG2, IGHG3, IGKC, IGKV4-1, IGLC2, KRT1, PROS1, SERPING1, VTN]

GO ID	GO Term	GO Levels	Nr. Genes	% Associated Genes	Term p -Value Corrected with Bonferroni step down	Associated Genes Found
GO:0006957	complement activation, alternative pathway	[4, 5, 6, 7, 8]	7	36.842106	9.60E-09	[C3, C8B, C9, CFB, CFD, CFH, CFHR5]
GO:0001867	complement activation, lectin pathway	[4, 5, 6, 7, 8]	4	44.444443	1.17E-04	[A2M, FCN3, KRT1, SERPING1]
GO:0006952	defense response	[3]	60	3.4843206	5.26E-30	[A2M, AHSG, APOA1, APOA2, APOA4, APOE, APOL1, C1QA, C1QB, C1QC, C1R, C3, C4A, C4B, C6, C8B, C9, CFB, CFD, CFH, CFHR5, CLU, F12, F2, FCN3, FGA, FGB, FN1, HP, HPR, HPX, HRG, IGHG1, IGHG2, IGHG3, IGHM, IGJ, IGKC, IGKV4-1, IGLC2, ITIH4, KLKB1, KNG1, KRT1, LBP, LTF, PF4, PPBP, PROS1, S100A8, S100A9, SAA4, SEPP1, SERPINA1, SERPINC1, SERPINF2, SERPING1, SNCA, THBS1, VTN]
GO:0006897	endocytosis	[5]	31	4.223433	3.48E-15	[AHSG, ALB, AMBP, APOA1, APOA5, APOB, APOC1, APOC2, APOE, APOL1, C3, C4A, C4B, COLEC11, FCN3, HBB, HP, HPR, HPX, IGHA1, IGHG1, IGHG2, IGHG3, IGKC, IGKV4-1, IGLC2, LBP, PRG4, SNCA, THBS1, VTN]
GO:0051649	establishment of localization in cell	[3]	33	1.3398294	4.59E-03	[A2M, ALB, ANG, APOA1, APOA2, APOE, CDH1, CFD, CLU, F2, F5, FGA, FGB, FGG, FN1, HRG, IGF1, IGF2, KNG1, MMRN1, PF4, PLG, PPBP, PROS1, S100A8, S100A9, SEPP1, SERPINA1, SERPINF2, SERPING1, SNCA, TF, THBS1]
GO:0006887	exocytosis	[4, 5, 6, 7]	26	6.5656567	6.12E-17	[A2M, ALB, APOA1, CFD, CLU, F5, FGA, FGB, FGG, FN1, HRG, IGF1, IGF2, KNG1, MMRN1, PF4, PLG, PPBP, PROS1, SEPP1, SERPINA1, SERPINF2, SERPING1, SNCA, TF, THBS1]
GO:0030198	extracellular matrix organization	[4]	14	3.5264482	1.02E-04	[A2M, CDH1, FGA, FGB, FGG, FN1, KLKB1, PLG, SERPINF2, SNCA, TGFBI, THBS1, TTR, VTN]
GO:0042730	fibrinolysis	[5, 6, 7, 8, 9]	12	42.857143	3.38E-17	[APOH, F11, F12, F2, HRG, KLKB1, KRT1, PLG, PROS1, SERPINF2, SERPING1, THBS1]
GO:0046503	glycerolipid catabolic process	[5, 6]	8	17.777779	2.04E-07	[APOA1, APOA2, APOA4, APOA5, APOB, APOC1, APOC2, APOE]
GO:0046486	glycerolipid metabolic process	[4, 5]	12	3.4482758	1.03E-03	[ANG, APOA1, APOA2, APOA4, APOA5, APOB, APOC1, APOC2, APOE, APOH, C3, PON1]
GO:0034380	high-density lipoprotein particle assembly	[4, 5, 7]	5	45.454544	2.61E-06	[APOA1, APOA2, APOA4, APOA5, APOE]
GO:0034384	high-density lipoprotein particle clearance	[4, 5]	4	40	1.93E-04	[APOA1, APOA2, APOC2, APOE]
GO:0034375	high-density lipoprotein particle remodeling	[4, 5, 7]	5	31.25	2.36E-05	[APOA1, APOA2, APOA4, APOC1, APOE]
GO:0042592	homeostatic process	[3]	28	1.8555335	5.18E-05	[ALB, ANG, APOA1, APOA2, APOA4, APOA5, APOB, APOC2, APOC4, APOE, CDH1, CFH, F2, HPX, IGF1, IGFBP5, IGHA1, IGHG3, IGJ, IGKC, KNG1, KRT1, LTF, PLG, S100A8, S100A9, SNCA, TF]
GO:0006959	humoral immune response	[3]	31	11.071428	9.21E-28	[A2M, C1QA, C1QB, C1QC, C1R, C3, C4A, C4B, C6, C8B, C9, CFB, CFD, CFH, CFHR1, CFHR5, CLU, FCN3, HPX, IGHG1, IGHG2, IGHG3, IGHM, IGKC, IGKV4-1, IGLC2, KRT1, LTF, PROS1, SERPING1, VTN]
GO:0002455	humoral immune response mediated by circulating immunoglobulin	[4, 7]	19	10.326087	2.07E-15	[C1QA, C1QB, C1QC, C1R, C3, C4A, C4B, C6, C8B, C9, CLU, HPX, IGHG1, IGHG2, IGHG3, IGKC, IGKV4-1, IGLC2, SERPING1]

GO ID	GO Term	GO Levels	Nr. Genes	% Associated Genes	Term p -Value Corrected with Bonferroni step down	Associated Genes Found
GO:0042744	hydrogen peroxide catabolic process	[3, 4, 5]	5	18.518518	4.02E-04	[APOA4, HBB, HP, HPR, SNCA]
GO:0006954	inflammatory response	[4]	36	5.590062	2.53E-22	[A2M, AHSG, APOA1, APOA2, APOE, C3, C4A, C4B, C6, C8B, C9, CFB, CFH, F12, F2, FN1, HP, HPR, ITIH4, KLKB1, KNG1, KRT1, LBP, PF4, PPBP, PROS1, S100A8, S100A9, SAA4, SEPP1, SERPINA1, SERPINC1, SERPINF2, SERPING1, THBS1, VTN]
GO:0045087	innate immune response	[3, 4]	40	3.6968577	1.35E-18	[A2M, APOA4, APOL1, C1QA, C1QB, C1QC, C1R, C3, C4A, C4B, C6, C8B, C9, CFB, CFD, CFH, CFHR5, CLU, F12, FCN3, FGA, FGB, HPX, IGHG1, IGHG2, IGHG3, IGHM, IGJ, IGKC, IGKV4-1, IGLC2, KRT1, LBP, LTF, PROS1, S100A8, S100A9, SERPING1, SNCA, VTN]
GO:0050900	leukocyte migration	[2, 4, 5, 6]	12	3.5928144	6.78E-04	[APOB, F2, FN1, LBP, PF4, PLG, PPBP, PROS1, S100A8, S100A9, SEPP1, THBS1]
GO:0002523	leukocyte migration involved in inflammatory response	[3, 5, 6, 7]	4	26.666666	1.18E-03	[LBP, PPBP, S100A8, S100A9]
GO:0055088	lipid homeostasis	[5]	8	6.9565215	3.74E-04	[APOA1, APOA2, APOA4, APOA5, APOB, APOC2, APOC4, APOE]
GO:0010876	lipid localization	[3]	20	5.8139534	1.78E-11	[ALB, APOA1, APOA2, APOA4, APOA5, APOB, APOC1, APOC2, APOC4, APOE, APOH, APOL1, C3, CLU, LBP, PON1, S100A8, S100A9, SERPINA5, THBS1]
GO:0006629	lipid metabolic process	[3]	22	1.6666666	9.15E-03	[ALB, ANG, APOA1, APOA2, APOA4, APOA5, APOB, APOC1, APOC2, APOC4, APOE, APOH, APOL1, C3, CLU, IGF2, IGFBP7, PON1, S100A8, S100A9, SNCA, TTR]
GO:0042157	lipoprotein metabolic process	[4, 5]	11	8.088235	5.25E-07	[ALB, APOA1, APOA2, APOA4, APOA5, APOB, APOC1, APOC2, APOE, APOL1, PON1]
GO:0043170	macromolecule metabolic process	[3]	82	0.91917944	2.19E-05	[A2M, AHSG, ALB, AMBP, ANG, APOA1, APOA2, APOA4, APOA5, APOB, APOC1, APOC2, APOE, APOH, APOL1, BNC2, C1QA, C1QB, C1QC, C1R, C3, C4A, C4B, C6, C8B, C9, CDH1, CFB, CFD, CFH, CFHR1, CFHR5, CLU, COLEC11, CPN1, F11, F12, F2, F5, FCN3, FGA, FN1, HP, HPR, HPX, HRG, IGF1, IGF2, IGFBP2, IGFBP3, IGFBP5, IGHA1, IGHG1, IGHG2, IGHG3, IGKC, IGKV4-1, IGLC2, ITIH2, ITIH4, KLKB1, KNG1, KRT1, LBP, LTF, PF4, PLG, PON1, PRG4, PROS1, RNASE4, S100A8, S100A9, SERPINA1, SERPINA5, SERPINC1, SERPIND1, SERPINF2, SERPING1, SNCA, THBS1, VTN]
GO:0042116	macrophage activation	[4, 6]	6	11.764706	4.66E-04	[CLU, LBP, S100A8, S100A9, SNCA, THBS1]
GO:2001234	negative regulation of apoptotic signaling pathway	[4, 5, 6, 7, 8]	9	3.982301	6.96E-03	[CLU, FGA, FGB, FGG, IGF1, PF4, S100A8, S100A9, THBS1]
GO:0048519	negative regulation of biological process	[1, 2, 3]	60	1.3767784	3.85E-09	[A2M, AHSG, AK1, ALB, AMBP, ANG, APOA1, APOA2, APOA4, APOC1, APOC2, APOE, APOH, ASPN, C1QC, C3, C4A, C4B, CDH1, CFH, CLU, F11, F12, F2, FCN3, FGA, FGB, FGG, GSN, HP, HPR, HRG, IGF1, IGFBP3, IGFBP5, IGFBP7, ITIH2, ITIH4, KLKB1, KNG1, KRT1, LBP, LTF, PF4, PLG, PON1, PRG4, PROS1, S100A8, S100A9, SERPINA1, SERPINA5, SERPINC1, SERPIND1, SERPINF2, SERPING1, SNCA, TGFBI, THBS1, VTN]
GO:0030195	negative regulation of blood coagulation	[4, 5, 6, 7, 8]	16	31.37255	6.13E-21	[APOE, APOH, F11, F12, F2, FGA, HRG, KLKB1, KNG1, KRT1, PLG, PROS1, SERPINF2, SERPING1,THBS1, VTN]
GO:0043086	negative regulation of catalytic activity	[3, 4, 5]	28	3.482587	2.55E-11	[A2M, AHSG, AMBP, APOA1, APOA2, APOC1, APOC2, APOE, C3, C4A, C4B, HP, HPR, HRG, ITIH2, ITIH4, KNG1, LTF, PROS1, SERPINA1, SERPINA5, SERPINC1, SERPIND1, SERPINF2, SERPING1, SNCA, THBS1, VTN]

GO ID	GO Term	GO Levels	Nr. Genes	% Associated Genes	Term p -Value Corrected with Bonferroni step down	Associated Genes Found
GO:0031324	negative regulation of cellular metabolic process	[3, 4, 5]	36	1.6933208	4.00E-06	[A2M, AHSG, AMBP, ANG, APOA4, APOC1, APOE, C3, C4A, C4B, CLU, F2, FCN3, HP, HPR, HRG, IGF1, IGFBP3, IGFBP5, ITIH2, ITIH4, KNG1, PON1, PRG4, PROS1, S100A8, S100A9, SERPINA1, SERPINA5, SERPINC1, SERPIND1, SERPINF2, SERPING1, SNCA, THBS1, VTN]
GO:0048523	negative regulation of cellular process	[2, 3, 4]	55	1.3729405	8.46E-08	[A2M, AHSG, AK1, ALB, AMBP, ANG, APOA1, APOA2, APOA4, APOC1, APOC2, APOE, APOH, ASPNC1QC, C3, C4A, C4B, CDH1, CFH, CLU, F2, FCN3, FGA, FGB, FGG, GSN, HP, HPR, HRG, IGF1, IGFBP3, IGFBP5, IGFBP7, ITIH2, ITIH4, KNG1, LTF, PF4, PLG, PON1, PRG4, PROS1, S100A8, S100A9, SERPINA1, SERPINA5, SERPINC1, SERPIND1, SERPINF2, SERPING1, SNCA, TGFBI, THBS1, VTN]
GO:0032269	negative regulation of cellular protein metabolic process	[4, 5, 6, 7]	27	3.3415842	2.15E-10	[A2M, AHSG, AMBP, ANG, APOE, C3, C4A, C4B, F2, HRG, IGFBP3, IGFBP5, ITIH2, ITIH4, KNG1, PROS1, S100A8, S100A9, SERPINA1, SERPINA5, SERPINC1, SERPIND1, SERPINF2, SERPING1, SNCA, THBS1, VTN]
GO:0032375	negative regulation of cholesterol transport	[5, 6, 7, 8, 9]	4	30.76923	6.36E-04	[APOA2, APOC1, APOC2, APOE]
GO:0010951	negative regulation of endopeptidase activity	[6, 7, 8, 9, 10]	20	8.230453	2.22E-14	[A2M, AHSG, AMBP, C3, C4A, C4B, HRG, ITIH2, ITIH4, KNG1, PROS1, SERPINA1, SERPINA5, SERPINC1, SERPIND1, SERPINF2, SERPING1, SNCA, THBS1, VTN]
GO:0051918	negative regulation of fibrinolysis	[2, 3, 4, 5, 6, 7, 8, 9]	6	54.545456	1.56E-08	[APOH, F2, HRG, PLG, SERPINF2, THBS1]
GO:0051346	negative regulation of hydrolase activity	[4, 5, 6]	24	6.3324537	4.49E-15	[A2M, AHSG, AMBP, APOA1, APOA2, APOC1, C3, C4A, C4B, HRG, ITIH2, ITIH4, KNG1, LTF, PROS1, SERPINA1, SERPINA5, SERPINC1, SERPIND1, SERPINF2, SERPING1, SNCA, THBS1, VTN]
GO:0002683	negative regulation of immune system process	[2, 3, 4]	11	3.2738094	4.60E-03	[A2M, AMBP, APOA1, APOA2, C1QC, LTF, PF4, S100A8, S100A9, SERPING1, THBS1]
GO:0032369	negative regulation of lipid transport	[3, 4, 5, 6, 7]	5	18.518518	4.02E-04	[APOA2, APOC1, APOC2, APOE, THBS1]
GO:0010605	negative regulation of macromolecule metabolic process	[3, 4, 5]	34	1.6528926	2.31E-05	[A2M, AHSG, AMBP, ANG, APOA4, APOE, C3, C4A, C4B, CLU, F2, FCN3, FGA, HRG, IGFBP3, IGFBP5, ITIH2, ITIH4, KNG1, PF4, PON1, PRG4, PROS1, S100A8, S100A9, SERPINA1, SERPINA5, SERPINC1, SERPIND1, SERPINF2, SERPING1, SNCA, THBS1, VTN]
GO:0009892	negative regulation of metabolic process	[2, 3, 4]	42	1.7449107	3.93E-08	[A2M, AHSG, AMBP, ANG, APOA1, APOA2, APOA4, APOC1, APOC2, APOE, C3, C4A, C4B, CLU, F2, FCN3, FGA, HP, HPR, HRG, IGF1, IGFBP3, IGFBP5, ITIH2, ITIH4, KNG1, LTF, PF4, PON1, PRG4, PROS1, S100A8, S100A9, SERPINA1, SERPINA5, SERPINC1, SERPIND1, SERPINF2, SERPING1, SNCA, THBS1, VTN]
GO:0051241	negative regulation of multicellular organismal process	[2, 3, 4]	29	3.128371	1.22E-10	[AHSG, APOA1, APOA2, APOC1, APOC2, APOE, APOH, ASPN, C1QC, F11, F12, F2, FGA, HRG, IGF1, IGFBP5, KLKB1, KNG1, KRT1, LBP, LTF, PF4, PLG, PRG4, PROS1, SERPINF2, SERPING1, THBS1, VTN]
GO:0051248	negative regulation of protein metabolic process	[4, 5, 6]	32	3.6281178	7.24E-14	[A2M, AHSG, AMBP, ANG, APOA4, APOE, C3, C4A, C4B, CLU, F2, FGA, HRG, IGFBP3, IGFBP5, ITIH2, ITIH4, KNG1, PON1, PRG4, PROS1, S100A8, S100A9, SERPINA1, SERPINA5, SERPINC1, SERPIND1, SERPINF2, SERPING1, SNCA, THBS1, VTN]
GO:0045861	negative regulation of proteolysis	[5, 6, 7, 8]	21	6.1764708	1.11E-12	[A2M, AHSG, AMBP, C3, C4A, C4B, F2, HRG, ITIH2, ITIH4, KNG1, PROS1, SERPINA1, SERPINA5, SERPINC1, SERPIND1, SERPINF2, SERPING1, SNCA, THBS1, VTN]
GO:0032102	negative regulation of response to external stimulus	[3, 4, 5]	19	7.949791	2.82E-13	[APOA1, APOE, APOH, F11, F12, F2, FGA, HRG, KLKB1, KNG1, KRT1, LTF, PLG, PROS1, SERPINC1, SERPINF2, SERPING1, THBS1, VTN]
GO:0048585	negative regulation of response to stimulus	[2, 3, 4]	37	2.820122	5.06E-13	[A2M, AHSG, AMBP, APOA1, APOA2, APOE, APOH, ASPN, CDH1, CLU, F11, F12, F2, FGA, FGB, FGG, HP, HPR, HRG, IGF1, IGFBP3, IGFBP5, KLKB1, KNG1, KRT1, LTF, PF4, PLG, PROS1, S100A8, S100A9, SERPINC1, SERPINF2, SERPING1, SNCA, THBS1, VTN]

GO ID	GO Term	GO Levels	Nr. Genes	% Associated Genes	Term p -Value Corrected with Bonferroni step down	Associated Genes Found
GO:1903035	negative regulation of response to wounding	[3, 4, 5, 6]	18	11.920529	1.27E-15	[APOA1, APOE, APOH, F11, F12, F2, FGA, HRG, KLKB1, KNG1, KRT1, PLG, PROS1, SERPINC1, SERPINF2, SERPING1, THBS1, VTN]
GO:0009968	negative regulation of signal transduction	[3, 4, 5, 6]	20	1.8656716	4.76E-03	[AHSG, AMBP, APOA1, APOE, ASPN, CDH1, CLU, FGA, FGB, FGG, HRG, IGF1, IGFBP3, IGFBP5, LTF, PF4, S100A8, S100A9, SNCA, THBS1]
GO:0006638	neutral lipid metabolic process	[4, 5]	12	9.302325	1.64E-08	[ANG, APOA1, APOA2, APOA4, APOA5, APOB, APOC1, APOC2, APOE, APOH, C3, SNCA]
GO:0015850	organic hydroxy compound transport	[3, 4, 5]	12	5.3333335	9.84E-06	[ALB, APOA1, APOA2, APOA4, APOA5, APOB, APOC1, APOC2, APOE, CLU, PON1, SNCA]
GO:0071702	organic substance transport	[4]	33	1.337115	4.77E-03	[ALB, ANG, APOA1, APOA2, APOA4, APOA5, APOB, APOC1, APOC2, APOC4, APOE, APOH, APOL1, C3, CDH1, CLU, FGA, FGB, FGG, HBB, HPX, HRG, IGF1, LBP, PON1, PPBP, S100A8, S100A9, SERPINA5, SNCA, TF, THBS1, TTR]
GO:0006909	phagocytosis	[3, 4, 5, 6]	13	3.8575668	1.06E-04	[AHSG, C3, C4A, C4B, FCN3, IGHG1, IGHG2, IGHG3, IGKC, IGKV4-1, IGLC2, LBP, THBS1]
GO:0033700	phospholipid efflux	[5, 6, 7, 8, 9]	7	53.846153	3.39E-10	[APOA1, APOA2, APOA4, APOA5, APOC1, APOC2, APOE]
GO:0007603	phototransduction, visible light	[5, 6, 7]	7	6.542056	2.84E-03	[APOA1, APOA2, APOA4, APOB, APOC2, APOE, TTR]
GO:0034381	plasma lipoprotein particle clearance	[3, 4]	6	18.75	2.66E-05	[APOA1, APOA2, APOB, APOC1, APOC2, APOE]
GO:0034369	plasma lipoprotein particle remodeling	[3, 4, 6]	8	29.62963	2.35E-09	[APOA1, APOA2, APOA4, APOA5, APOB, APOC1, APOC2, APOE]
GO:0031639	plasminogen activation	[6, 7]	6	33.333332	5.97E-07	[APOH, F11, F12, KLKB1, SERPINF2, THBS1]
GO:0030168	platelet activation	[4, 5, 6]	28	10.447762	4.88E-24	[A2M, ALB, APOA1, APOE, CFD, CLU, F2, F5, FGA, FGB, FGG, FN1, HBB, HRG, IGF1, IGF2, KNG1, MMRN1, PF4, PLG, PPBP, PROS1, SEPP1, SERPINA1, SERPINF2, SERPING1, TF, THBS1]
GO:0048518	positive regulation of biological process	[1, 2, 3]	76	1.4896119	1.64E-16	[A2M, AHSG, ALB, ANG, APOA1, APOA2, APOA4, APOA5, APOB, APOC1, APOC2, APOC4, APOE, APOH, C1QA, C1QB, C1QC, C1R, C3, C4A, C4B, C6, C8B, C9, CDH1, CFB, CFD, CFH, CFHR1, CFHR5, CLU, F11, F12, F2, FCN3, FGA, FGB, FGG, FN1, GSN, HBB, HP, HPR, HPX, HRG, IGF1, IGF2, IGFBP2, IGFBP3, IGFBP5, IGHA1, IGHG1, IGHG2, IGHG3, IGJ, IGKC, IGKV4-1, IGLC2, KLKB1, KNG1, KRT1, LBP, LTF, PF4, PLG, PON1, PPBP, PROS1, S100A8, S100A9, SEPP1, SERPINF2, SERPING1, SNCA, THBS1, VTN]
GO:0051130	positive regulation of cellular component organization	[2, 3, 4, 5]	21	2.4764152	2.80E-05	[AHSG, APOA1, APOA5, APOC2, APOE, C3, C4A, C4B, CLU, FGA, FGB, FGG, FN1, GSN, HRG, IGF1, IGF2, IGJ, SERPINF2, SNCA, VTN]
GO:0045807	positive regulation of endocytosis	[3, 4, 5, 6, 7]	7	7.2916665	1.40E-03	[AHSG, APOA5, C3, C4A, C4B, SNCA, VTN]
GO:0051919	positive regulation of fibrinolysis	[2, 3, 4, 5, 6, 7, 8, 9]	4	80	4.85E-06	[F11, F12, KLKB1, PLG]

GO ID	GO Term	GO Levels	Nr. Genes	% Associated Genes	Term p -Value Corrected with Bonferroni step down	Associated Genes Found
GO:0050778	positive regulation of immune response	[3, 4, 5]	34	4.7091413	1.79E-18	[A2M, C1QA, C1QB, C1QC, C1R, C3, C4A, C4B, C6, C8B, C9, CFB, CFD, CFH, CFHR1, CFHR5, CLU, FCN3, HPX, HRG, IGHG1, IGHG2, IGHG3, IGKC, IGKV4-1, IGLC2, KRT1, LBP, LTF, PROS1, S100A8, S100A9, SERPING1, VTN]
GO:0002684	positive regulation of immune system process	[2, 3, 4]	41	4.1922293	3.07E-21	[A2M, C1QA, C1QB, C1QC, C1R, C3, C4A, C4B, C6, C8B, C9, CFB, CFD, CFH, CFHR1, CFHR5, CLU, FCN3, HPX, HRG, IGF1, IGF2, IGFBP2, IGHG1, IGHG2, IGHG3, IGKC, IGKV4-1, IGLC2, KRT1, LBP, LTF, PF4, PPBP, PROS1, S100A8, S100A9, SEPP1, SERPING1, THBS1, VTN]
GO:1902533	positive regulation of intracellular signal transduction	[4, 5, 6, 7]	18	2.1791768	1.77E-03	[APOA1, CLU, F2, FGA, FGB, FGG, HPX, IGF1, IGF2, IGFBP3, IGFBP5, LTF, PF4, S100A8, S100A9, SEPP1, SERPINF2, THBS1]
GO:0046889	positive regulation of lipid biosynthetic process	[4, 5, 6, 7]	6	10	1.21E-03	[APOA1, APOA4, APOA5, APOC2, APOE, IGF2]
GO:0045834	positive regulation of lipid metabolic process	[3, 4, 5, 6]	8	6.779661	4.53E-04	[APOA1, APOA2, APOA4, APOA5, APOC1, APOC2, APOE, IGF2]
GO:0051240	positive regulation of multicellular organismal process	[2, 3, 4]	27	2.0626433	1.08E-05	[ALB, APOA2, APOA5, APOC2, APOE, APOH, C3, C6, CLU, F12, F2, FGA, FGB, FGG, FN1, HRG, IGF1, KNG1, LBP, LTF, PF4, PLG, S100A8, S100A9, SEPP1, SERPINF2, THBS1]
GO:0045937	positive regulation of phosphate metabolic process	[5, 6, 7]	21	2.1042085	4.16E-04	[ANG, APOC2, APOE, C3, CLU, F2, FGA, FGB, FGG, HPX, IGF1, IGF2, IGFBP3, LTF, PF4, S100A8, S100A9, SERPINF2, SNCA, THBS1, VTN]
GO:0042327	positive regulation of phosphorylation	[6, 7, 8]	18	2.0408163	4.36E-03	[ANG, C3, CLU, F2, FGA, FGB, FGG, HPX, IGF1, IGF2, IGFBP3, LTF, S100A8, S100A9, SERPINF2, SNCA, THBS1, VTN]
GO:0010954	positive regulation of protein processing	[5, 6, 7]	4	23.529411	2.01E-03	[C3, C6, F12, KLKB1]
GO:0048584	positive regulation of response to stimulus	[2, 3, 4]	54	2.721774	9.33E-21	[A2M, ALB, APOA1, APOH, C1QA, C1QB, C1QC, C1R, C3, C4A, C4B, C6, C8B, C9, CFB, CFD, CFH, CFHR1, CFHR5, CLU, F12, F2, FCN3, FGA, FGB, FGG, HPX, HRG, IGF1, IGF2, IGFBP3, IGFBP5, IGHG1, IGHG2, IGHG3, IGKC, IGKV4-1, IGLC2, KLKB1, KRT1, LBP, LTF, PF4, PLG, PPBP, PROS1, S100A8, S100A9, SEPP1, SERPINF2, SERPING1, SNCA, THBS1, VTN]
GO:1903036	positive regulation of response to wounding	[3, 4, 5, 6]	14	9.459459	2.55E-10	[APOH, C3, F12, F2, HRG, KLKB1, LBP, PLG, S100A8, S100A9, SEPP1, SERPINF2, THBS1, VTN]
GO:0051050	positive regulation of transport	[2, 3, 4, 5]	20	2.6455026	2.13E-05	[AHSG, ANG, APOA5, APOE, C3, C4A, C4B, CDH1, CFH, F2, FGA, FGB, FGG, IGF1, KNG1, PON1, S100A8, S100A9, SNCA, VTN]
GO:0090303	positive regulation of wound healing	[4, 5, 6, 7]	9	18.367348	1.11E-08	[APOH, F12, F2, HRG, PLG, SEPP1, SERPINF2, THBS1, VTN]
GO:0072376	protein activation cascade	[2, 3, 4, 5]	36	16.90141	8.58E-40	[A2M, APOH, C1QA, C1QB, C1QC, C1R, C3, C4A, C4B, C6, C8B, C9, CFB, CFD, CFH, CFHR1, CFHR5, CLU, F11, F12, F2, FCN3, FGA, IGHG1, IGHG2, IGHG3, IGKC, IGKV4-1, IGLC2, KLKB1, KNG1, KRT1, PROS1, SERPINC1, SERPING1, VTN]
GO:0019538	protein metabolic process	[3, 4]	79	1.5110941	2.79E-18	[A2M, AHSG, ALB, AMBP, ANG, APOA1, APOA2, APOA4, APOA5, APOB, APOC1, APOC2, APOE, APOH, APOL1, C1QA, C1QB, C1QC, C1R, C3, C4A, C4B, C6, C8B, C9, CDH1, CFB, CFD, CFH, CFHR1, CFHR5, CLU, COLEC11, CPN1, F11, F12, F2, F5, FCN3, FGA, FN1, HP, HPR, HPX, HRG, IGF1, IGF2, IGFBP2, IGFBP3, IGFBP5, IGHA1, IGHG1, IGHG2, IGHG3, IGKC, IGKV4-1, IGLC2, ITIH2, ITIH4, KLKB1, KNG1, KRT1, LBP, LTF, PLG, PON1, PRG4, PROS1, S100A8, S100A9, SERPINA1, SERPINA5, SERPINC1, SERPIND1, SERPINF2, SERPING1, SNCA, THBS1, VTN]
GO:0016485	protein processing	[4, 5]	32	5.3333335	8.46E-19	[A2M, AHSG, AMBP, APOH, C3, C4A, C4B, C6, C8B, C9, CFB, CFH, F11, F12, F2, HRG, ITIH2, ITIH4, KLKB1, KNG1, PROS1, S100A8, S100A9, SERPINA1, SERPINA5, SERPINC1, SERPIND1, SERPINF2, SERPING1, SNCA, THBS1, VTN]

GO ID	GO Term	GO Levels	Nr. Genes	% Associated Genes	Term p -Value Corrected with Bonferroni step down	Associated Genes Found
GO:0006508	proteolysis	[4, 5]	39	2.5776603	1.24E-12	[A2M, AHSG, AMBP, APOE, C1R, C3, C4A, C4B, CDH1, CFB, CFD, CLU, CPN1, F11, F12, F2, F5, FN1, HP, HPR, HRG, ITIH2, ITIH4, KLKB1, KNG1, LTF, PLG, PROS1, S100A8, S100A9, SERPINA1, SERPINA5, SERPINC1, SERPIND1, SERPINF2, SERPING1, SNCA, THBS1, VTN]
GO:0006898	receptor-mediated endocytosis	[6]	21	5.483029	1.18E-11	[ALB, AMBP, APOA1, APOA5, APOB, APOC1, APOC2, APOE, APOL1, COLEC11, HBB, HP, HPR, HPX, IGHA1, IGKC, IGKV4-1, IGLC2, PRG4, SNCA, VTN]
GO:0031099	regeneration	[3]	8	5.633803	1.77E-03	[APOA1, APOA2, APOA4, APOA5, APOE, GSN, IGF1, PLG]
GO:0002673	regulation of acute inflammatory response	[5, 6, 7]	14	18.421053	1.76E-14	[A2M, C3, C4A, C4B, C6, C8B, C9, CFB, CFH, F12, KLKB1, PROS1, SERPING1, VTN]
GO:0030193	regulation of blood coagulation	[4, 5, 6, 7]	18	19.78022	9.00E-20	[APOE, APOH, F11, F12, F2, FGA, HRG, KLKB1, KNG1, KRT1, PLG, PROS1, SEPP1, SERPINC1, SERPINF2, SERPING1, THBS1, VTN]
GO:0050878	regulation of body fluid levels	[3]	39	5.18617	2.39E-23	[A2M, ALB, APOA1, APOB, APOE, APOH, CDH1, CFD, CLU, F11, F12, F2, F5, FGA, FGB, FGG, FN1, HBB, HRG, IGF1, IGF2, KLKB1, KNG1, KRT1, MMRN1, PF4, PLG, PPBP, PROS1, SEPP1, SERPINA1, SERPINA5, SERPINC1, SERPIND1, SERPINF2, SERPING1, TF, THBS1, VTN]
GO:0050790	regulation of catalytic activity	[3, 4]	39	1.9145802	1.67E-08	[A2M, AHSG, AMBP, ANG, APOA1, APOA2, APOA4, APOA5, APOC1, APOC2, APOE, APOH, C3, C4A, C4B, CDH1, CLU, FN1, HP, HPR, HRG, IGF2, IGFBP3, ITIH2, ITIH4, KNG1, LTF, PROS1, S100A8, S100A9, SERPINA1, SERPINA5, SERPINC1, SERPIND1, SERPINF2, SERPING1, SNCA, THBS1, VTN]
GO:0030155	regulation of cell adhesion	[3, 4]	16	2.6229508	6.53E-04	[APOA1, CDH1, FGA, FGB, FGG, GSN, HRG, IGF1, IGF2, IGFBP2, KNG1, PLG, SERPINF2, TGFBI, THBS1, VTN]
GO:0010810	regulation of cell-substrate adhesion	[4, 5]	8	4.6242776	7.46E-03	[APOA1, FGA, FGB, FGG, HRG, PLG, THBS1, VTN]
GO:0051128	regulation of cellular component organization	[2, 3, 4]	33	1.7142857	1.66E-05	[AHSG, APOA1, APOA2, APOA5, APOC1, APOC2, APOE, C3, C4A, C4B, CFH, CLU, F2, FGA, FGB, FGG, FN1, GSN, HRG, IGF1, IGF2, IGFBP2, IGFBP3, IGFBP5, IGFBP7, IGJ, S100A8, S100A9, SEPP1, SERPINF2, SNCA, THBS1, VTN]
GO:0032268	regulation of cellular protein metabolic process	[4, 5, 6]	34	1.6707617	1.77E-05	[A2M, AHSG, AMBP, ANG, APOA1, APOE, C3, C4A, C4B, CLU, F2, FN1, HPX, HRG, IGF1, IGF2, IGFBP3, IGFBP5, ITIH2, ITIH4, KNG1, LTF, PROS1, S100A8, S100A9, SERPINA1, SERPINA5, SERPINC1, SERPIND1, SERPINF2, SERPING1, SNCA, THBS1, VTN]
GO:0032374	regulation of cholesterol transport	[6, 7, 8]	8	20	7.50E-08	[APOA1, APOA2, APOA4, APOA5, APOC1, APOC2, APOE, PON1]
GO:0031347	regulation of defense response	[4, 5]	24	4.217926	3.97E-11	[A2M, AHSG, APOA1, APOE, C3, C4A, C4B, C6, C8B, C9, CFB, CFH, F12, HPX, KLKB1, KRT1, LBP, LTF, PROS1, S100A8, S100A9, SERPINC1, SERPING1, VTN]
GO:0030100	regulation of endocytosis	[3, 4, 5, 6]	9	5	1.13E-03	[AHSG, APOA5, APOC1, APOC2, C3, C4A, C4B, SNCA, VTN]
GO:0051917	regulation of fibrinolysis	[2, 3, 6, 7, 8, 9, 10]	9	50	3.16E-13	[APOH, F11, F12, F2, HRG, KLKB1, PLG, SERPINF2, THBS1]
GO:0034114	regulation of heterotypic cell-cell adhesion	[5, 6]	4	25	1.56E-03	[APOA1, FGA, FGB, FGG]
GO:0002920	regulation of humoral immune response	[4, 5]	13	25.490196	2.56E-15	[A2M, C3, C4A, C4B, C6, C8B, C9, CFB, CFH, HPX, PROS1, SERPING1, VTN]

GO ID	GO Term	GO Levels	Nr. Genes	% Associated Genes	Term p -Value Corrected with Bonferroni step down	Associated Genes Found
GO:2000295	regulation of hydrogen peroxide catabolic process	[4, 5, 6, 7]	3	75	6.19E-04	[HP, HPR, SNCA]
GO:0051336	regulation of hydrolase activity	[4, 5]	33	3.2195122	6.95E-13	[A2M, AHSG, AMBP, ANG, APOA1, APOA2, APOA4, APOA5, APOC1, APOC2, APOH, C3, C4A, C4B, CDH1, FN1, HRG, ITIH2, ITIH4, KNG1, LTF, PROS1, S100A8, S100A9, SERPINA1, SERPINA5, SERPINC1, SERPIND1, SERPINF2, SERPING1, SNCA, THBS1, VTN]
GO:0002697	regulation of immune effector process	[3, 4]	18	5.9016395	3.09E-10	[A2M, APOA1, APOA2, C3, C4A, C4B, C6, C8B, C9, CFB, CFH, HPX, LBP, PROS1, S100A8, S100A9, SERPING1, VTN]
GO:0050776	regulation of immune response	[3, 4]	38	3.9014373	2.98E-18	[A2M, AMBP, APOA1, APOA2, C1QA, C1QB, C1QC, C1R, C3, C4A, C4B, C6, C8B, C9, CDH1, CFB, CFD, CFH, CFHR1, CFHR5, CLU, FCN3, HPX, HRG, IGHG1, IGHG2, IGHG3, IGKC, IGKV4-1, IGLC2, KRT1, LBP, LTF, PROS1, S100A8, S100A9, SERPING1, VTN]
GO:0002682	regulation of immune system process	[2, 3]	46	3.3117352	3.52E-20	[A2M, AMBP, APOA1, APOA2, C1QA, C1QB, C1QC, C1R, C3, C4A, C4B, C6, C8B, C9, CDH1, CFB, CFD, CFH, CFHR1, CFHR5, CLU, FCN3, HPX, HRG, IGF1, IGF2, IGFBP2, IGHG1, IGHG2, IGHG3, IGKC, IGKV4-1, IGLC2, KRT1, LBP, LTF, PF4, PPBP, PROS1, S100A8, S100A9, SEPP1, SERPING1,SNCA, THBS1, VTN]
GO:0050727	regulation of inflammatory response	[4, 5, 6]	22	8.058608	7.60E-16	[A2M, AHSG, APOA1, APOE, C3, C4A, C4B, C6, C8B, C9, CFB, CFH, F12, KLKB1, KRT1, LBP, PROS1, S100A8, S100A9, SERPINC1, SERPING1, VTN]
GO:0043567	regulation of insulin-like growth factor receptor signaling pathway	[4, 5, 6, 8, 9]	4	16.666666	8.48E-03	[IGF1, IGFBP2, IGFBP3, IGFBP5]
GO:0030300	regulation of intestinal cholesterol absorption	[5, 6, 7, 8, 9]	4	50	6.55E-05	[APOA1, APOA2, APOA4, APOA5]
GO:0060191	regulation of lipase activity	[5, 6]	9	7.7586207	2.80E-05	[ANG, APOA1, APOA2, APOA4, APOA5, APOC1, APOC2, APOH, SNCA]
GO:0046890	regulation of lipid biosynthetic process	[4, 5, 6]	10	7.874016	4.18E-06	[APOA1, APOA4, APOA5, APOB, APOC1, APOC2, APOE, C3, IGF2, IGFBP7]
GO:0050994	regulation of lipid catabolic process	[4, 5, 6]	6	12.765958	2.86E-04	[APOA1, APOA2, APOA4, APOA5, APOC1, APOC2]
GO:0019216	regulation of lipid metabolic process	[4, 5]	12	4.2704625	1.10E-04	[APOA1, APOA2, APOA4, APOA5, APOB, APOC1, APOC2, APOE, C3, IGF2, IGFBP7, SNCA]
GO:0032368	regulation of lipid transport	[4, 5, 6]	11	11.458333	1.18E-08	[APOA1, APOA2, APOA4, APOA5, APOC1, APOC2, APOE, PON1, S100A8, S100A9, THBS1]
GO:0051004	regulation of lipoprotein lipase activity	[6, 7]	6	40	1.64E-07	[APOA1, APOA4, APOA5, APOC1, APOC2, APOH]
GO:0032879	regulation of localization	[2, 3]	36	1.6981132	3.71E-06	[AHSG, ANG, APOA1, APOA2, APOA4, APOA5, APOB, APOC1, APOC2, APOC4, APOE, APOH, C3,C4A, C4B, CDH1, CFH, F2, FGA, FGB, FGG, HRG, IGF1, IGFBP3, IGFBP5, KNG1, LBP, PF4, PON1, PPBP, S100A8, S100A9, SEPP1, SNCA, THBS1, VTN]
GO:0019222	regulation of metabolic process	[2, 3]	65	1.0110437	2.53E-04	[A2M, AHSG, AMBP, ANG, APOA1, APOA2, APOA4, APOA5, APOB, APOC1, APOC2, APOE, APOH, BNC2, C3, C4A, C4B, C6, C8B, C9, CDH1, CFB, CFH, CLU, COLEC11, F12, F2, FCN3, FGA, FGB, FGG, FN1, HBB, HP, HPR, HPX, HRG, IGF1, IGF2, IGFBP3, IGFBP5, IGFBP7, IGHA1, IGJ, ITIH2, ITIH4, KLKB1, KNG1, LBP, LTF, PF4, PON1, PRG4, PROS1, S100A8, S100A9, SERPINA1, SERPINA5, SERPINC1, SERPIND1, SERPINF2, SERPING1, SNCA, THBS1, VTN]

GO ID	GO Term	GO Levels	Nr. Genes	% Associated Genes	Term p -Value Corrected with Bonferroni step down	Associated Genes Found
GO:0051239	regulation of multicellular organismal process	[2, 3]	43	1.8391788	3.50E-09	[AHSG, ALB, APOA1, APOA2, APOA4, APOA5, APOC1, APOC2, APOE, APOH, ASPN, C1QC, C3, C6, CDH1, CLU, F11, F12, F2, FGA, FGB, FGG, FN1, HRG, IGF1, IGFBP5, KLKB1, KNG1, KRT1, LBP, LTF, PF4, PLG, PRG4, PROS1, S100A8, S100A9, SEPP1, SERPINC1, SERPINF2, SERPING1, THBS1, VTN]
GO:0052547	regulation of peptidase activity	[5, 6, 7]	24	5.955335	1.83E-14	[A2M, AHSG, AMBP, C3, C4A, C4B, CDH1, FN1, HRG, ITIH2, ITIH4, KNG1, PROS1, S100A8, S100A9, SERPINA1, SERPINA5, SERPINC1, SERPIND1, SERPINF2, SERPING1, SNCA, THBS1, VTN]
GO:0019220	regulation of phosphate metabolic process	[5, 6]	26	1.7071569	8.27E-04	[AHSG, AMBP, ANG, APOA1, APOC1, APOC2, APOE, C3, CLU, F2, FGA, FGB, FGG, HPX, HRG, IGF1, IGF2, IGFBP3, LTF, PF4, S100A8, S100A9, SERPINF2, SNCA, THBS1, VTN]
GO:0042325	regulation of phosphorylation	[6, 7]	23	1.7597551	2.32E-03	[AHSG, AMBP, ANG, APOA1, APOE, C3, CLU, F2, FGA, FGB, FGG, HPX, HRG, IGF1, IGF2, IGFBP3, LTF, S100A8, S100A9, SERPINF2, SNCA, THBS1, VTN]
GO:0080090	regulation of primary metabolic process	[3, 4]	57	1.0337323	1.66E-03	[A2M, AHSG, AMBP, ANG, APOA1, APOA2, APOA4, APOA5, APOB, APOC1, APOC2, APOE, BNC2, C3, C4A, C4B, C6, C8B, C9, CDH1, CFB, CFH, CLU, COLEC11, F12, F2, FCN3, FGA, FN1, HPX, HRG, IGF1, IGF2, IGFBP3, IGFBP5, IGFBP7, ITIH2, ITIH4, KLKB1, KNG1, LBP, LTF, PF4, PON1, PRG4, PROS1, S100A8, S100A9, SERPINA1, SERPINA5, SERPINC1, SERPIND1, SERPINF2, SERPING1, SNCA, THBS1, VTN]
GO:2000257	regulation of protein activation cascade	[3, 4, 5, 6]	14	38.88889	1.22E-19	[A2M, C3, C4A, C4B, C6, C8B, C9, CFB, CFH, FGA, PROS1, SERPINC1, SERPING1, VTN]
GO:0051246	regulation of protein metabolic process	[4, 5]	48	2.1323855	2.60E-13	[A2M, AHSG, AMBP, ANG, APOA1, APOA2, APOA4, APOE, C3, C4A, C4B, C6, C8B, C9, CDH1, CFB, CFH, CLU, F12, F2, FGA, FN1, HPX, HRG, IGF1, IGF2, IGFBP3, IGFBP5, ITIH2, ITIH4, KLKB1, KNG1, LBP, LTF, PON1, PRG4, PROS1, S100A8, S100A9, SERPINA1, SERPINA5, SERPINC1, SERPIND1, SERPINF2, SERPING1, SNCA, THBS1, VTN]
GO:0070613	regulation of protein processing	[5, 6]	28	7.070707	2.48E-19	[A2M, AHSG, AMBP, C3, C4A, C4B, C6, C8B, C9, CFB, CFH, F12, F2, HRG, ITIH2, ITIH4, KLKB1, KNG1, PROS1, SERPINA1, SERPINA5, SERPINC1, SERPIND1, SERPINF2, SERPING1, SNCA, THBS1, VTN]
GO:0030162	regulation of proteolysis	[5, 6]	27	3.8243625	8.53E-12	[A2M, AHSG, AMBP, APOE, C3, C4A, C4B, CDH1, CLU, F2, FN1, HRG, ITIH2, ITIH4, KNG1, PROS1, S100A8, S100A9, SERPINA1, SERPINA5, SERPINC1, SERPIND1, SERPINF2, SERPING1, SNCA, THBS1, VTN]
GO:0032101	regulation of response to external stimulus	[3, 4]	35	5.162242	1.93E-20	[A2M, AHSG, APOA1, APOE, APOH, C3, C4A, C4B, C6, C8B, C9, CFB, CFH, F11, F12, F2, FGA, HRG, KLKB1, KNG1, KRT1, LBP, LTF, PF4, PLG, PPBP, PROS1, S100A8, S100A9, SEPP1, SERPINC1, SERPINF2, SERPING1, THBS1, VTN]
GO:0048583	regulation of response to stimulus	[2, 3]	66	1.8681008	4.50E-18	[A2M, AHSG, ALB, AMBP, APOA1, APOA2, APOE, APOH, ASPN, C1QA, C1QB, C1QC, C1R, C3, C4A, C4B, C6, C8B, C9, CDH1, CFB, CFD, CFH, CFHR1, CFHR5, CLU, F11, F12, F2, FCN3, FGA, FGB, FGG, HP, HPR, HPX, HRG, IGF1, IGF2, IGFBP2, IGFBP3, IGFBP5, IGHG1, IGHG2, IGHG3, IGKC, IGKV4-1, IGLC2, KLKB1, KNG1, KRT1, LBP, LTF, PF4, PLG, PPBP, PROS1, S100A8, S100A9, SEPP1, SERPINC1, SERPINF2, SERPING1, SNCA, THBS1, VTN]
GO:0080134	regulation of response to stress	[3, 4]	39	3.5198555	2.90E-17	[A2M, AHSG, AMBP, APOA1, APOE, APOH, C3, C4A, C4B, C6, C8B, C9, CFB, CFH, CLU, F11, F12, F2, FGA, HP, HPR, HPX, HRG, KLKB1, KNG1, KRT1, LBP, LTF, PLG, PROS1, S100A8, S100A9, SEPP1, SERPINC1, SERPINF2, SERPING1, SNCA, THBS1, VTN]
GO:1903034	regulation of response to wounding	[4, 5]	32	8.205129	1.36E-24	[A2M, AHSG, APOA1, APOE, APOH, C3, C4A, C4B, C6, C8B, C9, CFB, CFH, F11, F12, F2, FGA, HRG, KLKB1, KNG1, KRT1, LBP, PLG, PROS1, S100A8, S100A9, SEPP1, SERPINC1, SERPINF2, SERPING1, THBS1, VTN]
GO:0019218	regulation of steroid metabolic process	[5, 6]	9	10.4651165	2.01E-06	[APOA1, APOA2, APOA4, APOA5, APOB, APOC1, APOE, IGF2, IGFBP7]
GO:0051049	regulation of transport	[3, 4]	26	1.642451	1.67E-03	[AHSG, ANG, APOA1, APOA2, APOA4, APOA5, APOC1, APOC2, APOE, C3, C4A, C4B, CDH1, CFH, F2, FGA, FGB, FGG, IGF1, KNG1, PON1, S100A8, S100A9, SNCA, THBS1, VTN]

GO ID	GO Term	GO Levels	Nr. Genes	% Associated Genes	Term p -Value Corrected with Bonferroni step down	Associated Genes Found
GO:0090207	regulation of triglyceride metabolic process	[4, 5, 6, 7, 8]	5	17.857143	4.84E-04	[APOA1, APOA4, APOA5, APOC2, C3]
GO:0010901	regulation of very-low-density lipoprotein particle remodeling	[3, 4, 5, 6, 7, 9]	4	66.666664	1.44E-05	[APOA1, APOA2, APOA5, APOC2]
GO:0060627	regulation of vesicle-mediated transport	[3, 4, 5]	12	3.269755	1.77E-03	[AHSG, APOA5, APOC1, APOC2, C3, C4A, C4B, FGA, FGB, FGG, SNCA, VTN]
GO:0009617	response to bacterium	[3, 5]	13	2.8017242	3.55E-03	[APOB, HP, HPR, IGHM, LBP, LTF, PF4, PPBP, S100A8, S100A9, SEPP1, SERPINA1, SNCA]
GO:0010035	response to inorganic substance	[3]	19	4.328018	1.50E-08	[ALB, APOA4, APOB, CDH1, FGA, FGB, FGG, GSN, HBB, HP, HPR, HRG, IGFBP2, PON1, S100A8, S100A9, SERPINA1, SNCA, THBS1]
GO:0033993	response to lipid	[4]	19	2.4265645	1.86E-04	[APOA1, APOA2, APOB, APOE, CPN1, IGF2, IGFBP2, IGFBP7, LBP, LTF, PF4, PON1, PPBP, S100A8, S100A9, SEPP1, SERPINA1, SNCA, THBS1]
GO:0032496	response to lipopolysaccharide	[4, 5, 7]	10	3.4722223	7.34E-03	[APOB, LBP, LTF, PF4, PPBP, S100A8, S100A9, SEPP1, SERPINA1, SNCA]
GO:0010038	response to metal ion	[4]	13	4.3333335	2.76E-05	[ALB, CDH1, FGA, FGB, FGG, GSN, HRG, IGFBP2, S100A8, S100A9, SERPINA1, SNCA, THBS1]
GO:0010033	response to organic substance	[3]	36	1.375621	7.79E-04	[AHSG, ALB, ANG, APOA1, APOA2, APOA4, APOA5, APOB, APOE, ASPN, CDH1, CLU, CPN1, F12, FGB, GSN, HPX, HRG, IGF2, IGFBP2, IGFBP5, IGFBP7, ITIH4, LBP, LTF, PF4, PON1, PPBP, S100A8, S100A9, SEPP1, SERPINA1, SERPINF2, SNCA, THBS1, VTN]
GO:1901700	response to oxygen-containing compound	[3]	26	1.8531718	1.76E-04	[AHSG, APOA2, APOA4, APOB, APOE, CDH1, CLU, GSN, HBB, HP, HPR, IGF2, IGFBP2, IGFBP5, IGFBP7, LBP, LTF, PF4, PON1, PPBP, S100A8, S100A9, SEPP1, SERPINA1, SNCA, THBS1]
GO:0009611	response to wounding	[3]	52	4.92891	3.97E-32	[A2M, AHSG, ALB, APOA1, APOA5, APOB, APOE, APOH, C3, C4A, C4B, C6, C8B, C9, CFB, CFD, CFH, CLU, F11, F12, F2, F5, FGA, FGB, FGG, FN1, GSN, HBB, HRG, IGF1, IGF2, KLKB1, KNG1, KRT1, LBP, MMRN1, PF4, PLG, PPBP, PROS1, S100A8, S100A9, SEPP1, SERPINA1, SERPINA5, SERPINC1, SERPIND1, SERPINF2, SERPING1, TF, THBS1, VTN]
GO:0001523	retinoid metabolic process	[7, 8]	7	7.7777777	9.12E-04	[APOA1, APOA2, APOA4, APOB, APOC2, APOE, TTR]
GO:0043691	reverse cholesterol transport	[6, 7, 8]	7	36.842106	9.60E-09	[APOA1, APOA2, APOA4, APOA5, APOC2, APOE, CLU]
GO:0032940	secretion by cell	[3, 4, 5, 6]	30	3.1847134	2.63E-11	[A2M, ALB, ANG, APOA1, APOA2, CFD, CLU, F5, FGA, FGB, FGG, FN1, HRG, IGF1, IGF2, KNG1, MMRN1, PF4, PLG, PPBP, PROS1, S100A8, S100A9, SEPP1, SERPINA1, SERPINF2, SERPING1, SNCA, TF, THBS1]
GO:0044765	single-organism transport	[2, 3, 4]	61	1.5741936	3.45E-12	[A2M, AHSG, ALB, ANG, APOA1, APOA2, APOA4, APOA5, APOB, APOC1, APOC2, APOC4, APOE, APOH, APOL1, C3, C4A, C4B, CDH1, CFD, CFH, CLU, F2, F5, FCN3, FGA, FGB, FGG, FN1, HBA2, HBB, HPX, HRG, IGF1, IGF2, IGHG1, IGHG2, IGHG3, IGKC, IGKV4-1, IGLC2, KNG1, LBP, LTF, MMRN1, PF4, PLG, PON1, PPBP, PROS1, S100A8, S100A9, SEPP1, SERPINA1, SERPINA5, SERPINF2, SERPING1, SNCA, TF, THBS1, TTR]
GO:0008202	steroid metabolic process	[4]	11	3.6065574	1.85E-03	[ALB, APOA1, APOA2, APOA4, APOA5, APOB, APOC1, APOE, APOL1, IGF2, IGFBP7]

GO ID	GO Term	GO Levels	Nr. Genes	% Associated Genes	Term p -Value Corrected with Bonferroni step down	Associated Genes Found
GO:0006810	transport	[3]	69	1.498046	7.20E-14	[A2M, AHSG, ALB, AMBP, ANG, APOA1, APOA2, APOA4, APOA5, APOB, APOC1, APOC2, APOC4, APOE, APOH, APOL1, C3, C4A, C4B, CDH1, CFD, CFH, CLU, COLEC11, F2, F5, FCN3, FGA, FGB, FGG, FN1, GSN, HBA2, HBB, HP, HPR, HPX, HRG, IGF1, IGF2, IGHA1, IGHG1, IGHG2, IGHG3, IGKC, IGKV4-1, IGLC2, KNG1, LBP, LTF, MMRN1, PF4, PLG, PON1, PPBP, PRG4, PROS1, S100A8, S100A9, SEPP1, SERPINA1, SERPINA5, SERPINF2, SERPING1, SNCA, TF, THBS1, TTR, VTN]
GO:0070328	triglyceride homeostasis	[7]	5	14.285714	1.51E-03	[APOA1, APOA4, APOA5, APOC2, APOC4]
GO:0001944	vasculature development	[4, 5, 6, 7]	16	2.6272578	6.40E-04	[ANG, APOB, APOE, APOH, C3, C6, FN1, HRG, IGF1, KLKB1, KRT1, PF4, PLG, SERPINF2, TGFBI, THBS1]
GO:0034447	very-low-density lipoprotein particle clearance	[4, 5]	3	50	2.95E-03	[APOC1, APOC2, APOE]
GO:0034372	very-low-density lipoprotein particle remodeling	[5, 6, 8]	6	46.153847	5.71E-08	[APOA1, APOA2, APOA4, APOA5, APOC2, APOE]
GO:0016192	vesicle-mediated transport	[4]	54	3.69863	2.53E-27	[A2M, AHSG, ALB, AMBP, APOA1, APOA5, APOB, APOC1, APOC2, APOE, APOL1, C3, C4A, C4B, CFD, CLU, COLEC11, F5, FCN3, FGA, FGB, FGG, FN1, GSN, HBB, HP, HPR, HPX, HRG, IGF1, IGF2, IGHA1, IGHG1, IGHG2, IGHG3, IGKC, IGKV4-1, IGLC2, KNG1, LBP, MMRN1, PF4, PLG, PPBP, PRG4, PROS1, SEPP1, SERPINA1, SERPINF2, SERPING1, SNCA, TF, THBS1, VTN]
GO:0042060	wound healing	[4]	41	5.5330634	6.65E-26	[A2M, ALB, APOA1, APOA5, APOB, APOE, APOH, CFD, CLU, F11, F12, F2, F5, FGA, FGB, FGG, FN1, GSN, HBB, HRG, IGF1, IGF2, KLKB1, KNG1, KRT1, MMRN1, PF4, PLG, PPBP, PROS1, S100A8, SEPP1, SERPINA1, SERPINA5, SERPINC1, SERPIND1, SERPINF2, SERPING1, TF, THBS1, VTN]
GO:0031638	zymogen activation	[5, 6]	9	7.2580647	4.99E-05	[APOH, F11, F12, KLKB1, S100A8, S100A9, SERPINF2, SNCA, THBS1]
